# Traditional Chinese medicine for the treatment of diabetic kidney disease: A study-level pooled analysis of 44 randomized controlled trials

**DOI:** 10.3389/fphar.2022.1009571

**Published:** 2022-10-13

**Authors:** Xuele Liu, Minyao Ge, Xinyu Zhai, Yang Xiao, Yaheng Zhang, Ziling Xu, Zhiguang Zhou, Zubing Mei, Xuejun Yang

**Affiliations:** ^1^ Institute of Nephrology, Shuguang Hospital Affiliated to Shanghai University of Traditional Chinese Medicine, Shanghai, China; ^2^ Department of Urology, Shuguang Hospital, Shanghai University of Traditional Chinese Medicine, Shanghai, China; ^3^ The National Clinical Research Center for Metabolic Diseases, Department of Metabolism and Endocrinology, The Second Xiangya Hospital, Central South University, Changsha, Hunan, China; ^4^ Department of Anorectal Surgery, Shuguang Hospital, Shanghai University of Traditional Chinese Medicine, Shanghai, China; ^5^ Anorectal Disease Institute of Shuguang Hospital, Shanghai, China

**Keywords:** traditional Chinese medicine, diabetic kidney disease (DKD), pooled analysis, randomized controlled trials (RCT), clinical efficacy

## Abstract

**Background:** Accumulating evidence suggests that traditional Chinese medicine (TCM) has significant effects on reducing 24-h urinary protein (24-h UPRO) and improves renal function indices. The current level of evidence-based medicine is still not enough due to the limitation of clinical center size and sample size.

**Objective:** We aimed to update the current evidence on the efficacy of TCM in the treatment of diabetic kidney disease (DKD).

**Methods:** PubMed, Embase, the Cochrane Library, and SinoMed were searched to identify randomized controlled trials (RCTs) comparing the clinical efficacy of TCM combined with Western medicine with that of Western medicine alone for the treatment of DKD. The main outcome measure was 24-h UPRO. The secondary outcomes were serum creatinine (Scr), blood urea nitrogen (BUN), glycosylated hemoglobin (HbA1c), fasting blood glucose (FBG), total cholesterol (TC), and triglyceride (TG). Meta-analyses were performed using random-effects models. The revised Cochrane risk-of-bias tool was used to assess the risk of bias.

**Results:** A total of 44 RCTs with 3,730 participants were included. The summary estimates showed that compared with Western medicine alone, TCM combined with Western medicine significantly improved 24-h UPRO [standardized mean difference (SMD) −1.10, 95% confidence interval (CI) −1.45 to −0.74]. Moreover, TCM combined with Western medicine significantly reduced the levels of other renal function indices, including Scr (SMD −1.25, 95% CI: −1.69 to −0.81) and BUN (SMD −0.75, 95% CI: −1.10 to −0.40). TCM combined with Western medicine also showed greater benefits in reducing the levels of FBG (SMD −0.31, 95% CI: −0.47 to −0.15) and HbA1c (SMD −0.62, 95% CI: −0.89 to −0.36) in patients with DKD. In addition, superior effects on the lipid profile were noted in the TCM combined with Western medicine group in terms of TG (SMD −1.17, 95% CI: −1.76 to −0.59) and TC (SMD −0.95, 95% CI: −1.43 to −0.47). The risk of bias could have resulted from selective reports, unclear randomization methods, unblinded assignments, and some missing data.

**Conclusion:** The results of this meta-analysis suggest that TCM combined with Western medicine has significant effects on reducing 24-h UPRO and improves renal function indices and lipid profiles compared with Western medicine alone for DKD. However, the results should be interpreted with caution due to the risk of bias of the included trials.

**Systematic Review Registration:** [https://www.crd.york.ac.uk/prospero/display_record.php?RecordID=213199], identifier [CRD: 42020213199].

## 1 Introduction

Globally, more than five million people die each year because they do not have access to critical treatment for kidney disease, and chronic kidney disease (CKD) is expected to be the fifth leading cause of death in the world by 2040. Approximately 30%–50% of the end-stage renal disease (ESRD) cases in the world are caused by diabetic kidney disease (DKD) (Ruiz-Ortega et al., 2020; Bakris et al., 2021). DKD has become the leading cause of ESRD in middle-aged and elderly individuals in China, and it is increasing worldwide at an alarming rate (KDOQI, 2007; Bourassa-Moreau et al., 2020). It is estimated that by 2035, the number of DKD patients will exceed 350 million (Gheith et al., 2016). The first symptom of DKD is microalbuminuria; with the progression of the disease, renal function continues to be impaired, and continuous microalbuminuria develops to massive albuminuria and eventually develops into ESRD (Afkarian et al., 2016; Chen et al., 2017; Wang et al., 2019). Proteinuria is the main independent risk factor for the progression of DKD (Hemmelgarn et al., 2010). At present, the treatment for reducing albuminuria in modern medicine is to administer drugs to control blood sugar and lower blood pressure (Emdin et al., 2015; Unger et al., 2020), including renin angiotensin aldosterone system (RAAS) blockers (Viberti et al., 1994; Haller et al., 2011; Unger et al., 2020), on the basis of lifestyle intervention (Perl et al., 2017; American Diabetes Association, 2019a). Drugs that can lower blood sugar and may reduce urinary protein to protect renal function include glucose cotransporter 2 inhibitors (SGLT2is) (Garofalo et al., 2019; Jardine et al., 2019; Heerspink et al., 2020), glucagon-like peptide-1 (GLP-1) receptor agonists (Marso et al., 2016; Mann et al., 2017; Muskiet et al., 2018; Pratley et al., 2019), and dipeptidyl peptidase (DPP)-4 inhibitors (Cornel et al., 2016; Groop et al., 2017; Mosenzon et al., 2017). However, the above methods cannot control the proteinuria of all patients with DKD.

In recent years, the field of traditional Chinese medicine (TCM) has represented a vast untapped resource for modern medicine. Researchers have begun to recognize TCM as a potential source of new drug candidates (Li and Zhang, 2008; Hu et al., 2017; Du et al., 2018). TCM acts on multiple targets through different signaling pathways to delay the progression of diseases (Gene Ontology Consortium, 2015). A large number of randomized controlled trials (RCTs) have shown that TCM combined with Western medicine in the treatment of DKD can better reduce urinary protein excretion and protect renal function (Tu et al., 2015; Wen et al., 2017). Mahuang Fuzi Shenzhuo decoction can enhance podocyte autophagy, inhibit the activation of the Wnt/*β*-Catenin signaling pathway stimulated by high glucose, and help to reduce podocyte injury in rats with DKD (Dai et al., 2020). The TCM capsule for replenishing qi and nourishing yins could significantly reduce the 24-h urinary albumin and the expression of CD34 and CD144 in the kidneys of DKD model rats and improve the pathological changes in glomerular hypertrophy, mesenteric matrix thickening, mesenteric thickening, and nodular hyperplasia (Zhou et al., 2019).

However, the current level of evidence-based medicine is still not enough due to the limitation of clinical center size and sample size. The evaluation and comparison of various treatment methods are not sufficient, and to the best of our knowledge, there is no comprehensive evaluation of the clinical efficacy of TCM combined with Western medicine [angiotensin-converting–enzyme inhibitor (ACEI)/angiotensin II receptor blocker (ARB)] in the treatment of DKD proteinuria under the guidance of different treatment methods.

The purpose of this meta-analysis is to provide a sufficient basis for the clinical application of TCM combined with Western medicine. We hope that the results of the study will provide clinicians with the best choice for the treatment of DKD proteinuria and provide them with a research direction.

## 2 Methods

This systematic review was guided by the recommendations for performing systematic reviews in the Cochrane Handbook, and the reporting was performed according to the Preferred Reporting Items for Systematic Reviews and Meta-Analyses (PRISMA) guidelines. The review protocol was registered with PROSPERO before commencement (CRD: 42020213199).

### 2.1 Information sources and searches

Two independent reviewers searched through major databases, including PubMed, EMBASE, the Cochrane Library and Chinese Bio-Medical, from their initiation through March 2020 and updated in December 2021 using the detailed search strategy and specific terms [(traditional Chinese medicine or herbal medicine or Chinese herbal drug) and (diabetic kidney disease or diabetic nephropathy or diabetic nephrosclerosis or diabetic glomerulosclerosis) and (randomized controlled trial or controlled clinical trial or randomized or placebo)], which were searched as free text words and as MeSH/Entree terms. Supplementary Material shows the detailed search strategy for each database. In addition, the references of the retrieved trials and previous related systematic reviews were also manually reviewed to identify potential missing eligible trials.

### 2.2 Study selection and eligibility criteria

Original studies were reviewed, and data abstraction was conducted by two independent authors (XL and YX). A group discussion was carried out for any discrepancies during this step until consensus was achieved. A senior author (ZM) was consulted to obtain a confirming suggestion. When necessary, we contacted the corresponding authors of the original studies for detailed information.

Studies were considered appropriate and were included in the analysis if they satisfied the following established inclusion criteria.

(1) Adult participants aged at least 18 years were diagnosed with DKD or clinical DKD according to its diagnostic (Alicic et al., 2017; Anders et al., 2018) and staging criteria established by American Diabetes Association in 2020 the National Kidney Foundation Disease Outcomes Quality Initiative (KDOQI) guidelines36 and Mogensen staging (Mogensen et al., 1983). No restrictions were applied on the age, sex, ethnicity, region or economic status of the included participants.
(2) Patients in the treatment group were treated with TCM combined with Western medicine, while patients in the control group were treated with Western medicine alone. The treatment dose, duration and frequency were not limited. In addition, patients in both groups received the same routine treatment, including the integrated management of blood pressure and nutrition, as recommended by the clinical practice guidelines for chronic kidney disease (American Diabetes Association, 2019a), (American Diabetes Association, 2019b). Patients with nondiabetic proteinuria who had ESRD or who received renal replacement therapy were excluded from the study.
(3) Western medicine alone was used as a common comparator for this meta-analysis.
(4) Trials were included that evaluated at least one of the following outcomes. We selected 24-h urinary protein (24-UPRO) as the primary outcome measure because it was one of the major measurements used to diagnose CKD and other kidney diseases and was also commonly reported as the primary outcome in the literature. The secondary outcomes included protein and renal function indicators [including serum creatinine (Scr) and blood urea nitrogen (BUN)], fasting blood glucose (FBG), glycosylated hemoglobin (HbA1c), triglyceride (TG), and total cholesterol (TC).
(5) RCTs were included regardless of blinding. We did not apply date and language restrictions.

### 2.3 Data collection and quality assessment

For each trial, the following details concerning the PICOS characteristics were abstracted: first author, year of publication, patient age, sample size, interventions, and chief outcome indicators obtained from the study.

Two authors independently assessed the risk of bias for each RCT according to the recommendation criteria of the Cochrane Handbook for Systematic Reviews of Interventions (Cumpston et al., 2019). There were seven domains that were evaluated, including random sequence generation, allocation concealment, blinding methods (including investigators, participants, and outcome assessment), attrition bias, reporting bias and other sources of bias. Each potential source of bias was evaluated at three levels: high, low or unclear bias. Any disagreements between the two authors were resolved through discussion.

### 2.4 Data synthesis and analysis

All statistical analyses were carried out with Review Manager Software (version 5.3, Cochrane Community, UK). We used continuous variables in this meta-analysis to pool the effect estimates [standardized mean difference (SMD)] using the generic inverse variance method. All analyses were performed using a more conservative random‐effects model. Leave-one-out sensitivity analysis was applied to assess the stability of the overall effect estimates. The intertrial heterogeneity was assessed with I‐square and chi‐square tests, with I^2^ > 50% indicating significant heterogeneity. For the main outcome, we also conducted subgroup analyses to explore the potential sources of heterogeneity based on the baseline characteristics of the included RCTs. Publication bias was tested by funnel plots and Egger’s test when the number of included studies was more than 10 for the studied outcome. A value of *p* < 0.05 was considered statistically significant.

## 3 Results

### 3.1 Study selection

**FIGURE 1:**
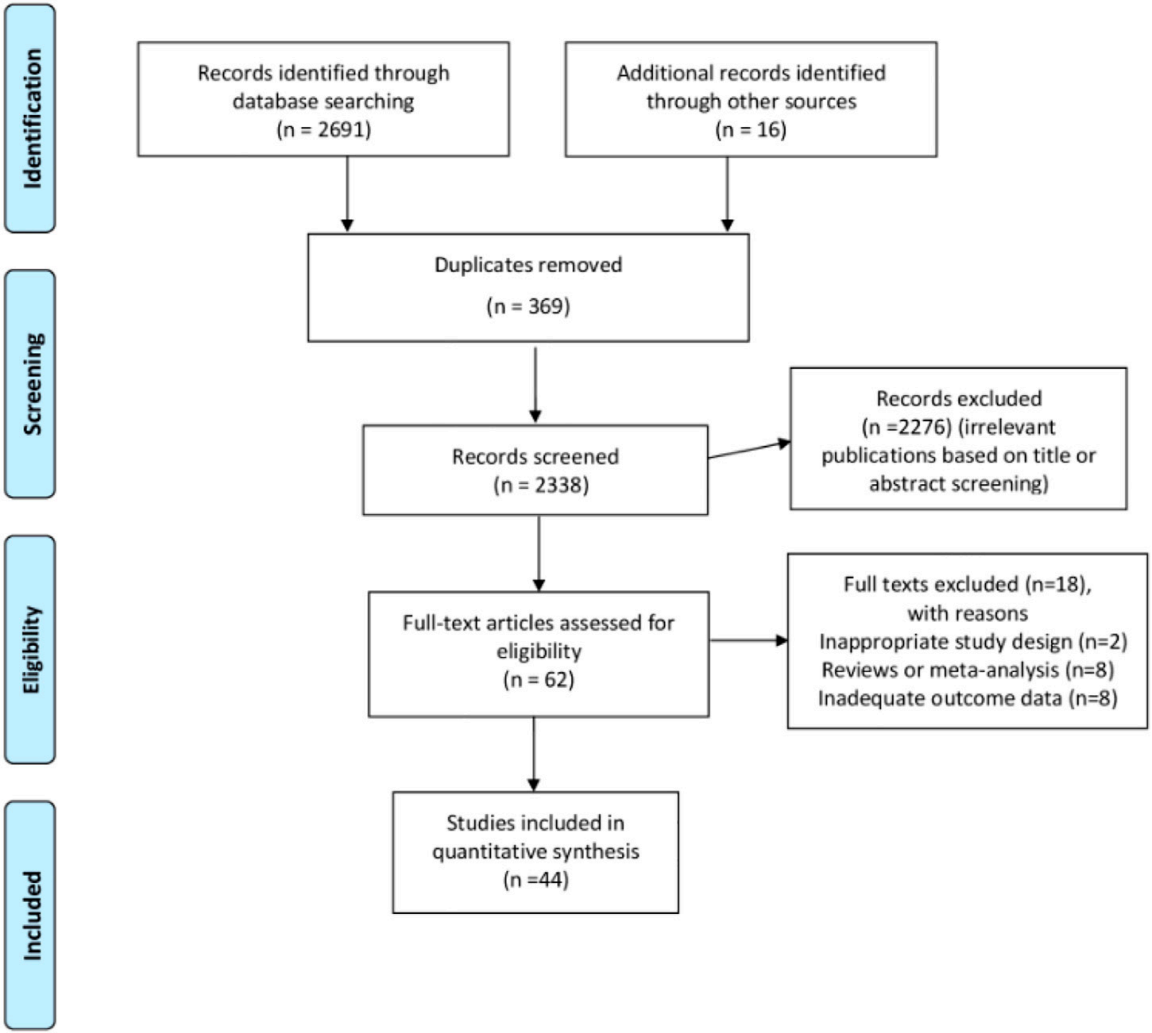
Flow diagram of study selection.

We present the flowchart of the literature search process in Figure 1. In summary, a total of 2,707 records were identified from the initial literature search. Our primary search strategy from the four major databases yielded 2,691 articles, including 265 records in PubMed, 409 records in Embase, 345 records in the Cochrane Library and 1,672 records in Chinese Bio-Medical. Moreover, 16 records were added through a manual reference search of related systematic reviews and original studies. EndNote X7 software was used to remove duplicate records, and 62 records remained for full-text review after we further removed unrelated records through title and abstract screening. During the process, 1,184 records were excluded due to studies being irrelevant to the effects of TCM on proteinuria in DKD patients. We carefully conducted the full-text review of the remaining 62 articles. Of these, 18 studies were excluded for multiple reasons, and 44 RCTs including 3,730 participants were finally included in the meta-analysis for the evaluation of TCM combined with Western medicine in the treatment of proteinuria in patients with DKD.

### 3.2 Study characteristics

All 44 RCTs [39-83] included in this study investigated TCM combined with Western medicine in the treatment of proteinuria. All the articles were sourced from Chinese publications, and the trials were all conducted in China between 2012 and 2019. Overall, the combined patient sample size was 203, ranging from 45 to 214. The basic characteristics of the trial patients and controls as well as the interventions of the 44 eligible studies are displayed in Table 1. Summary results of the outcome measures are shown in Table 2. Studies of composition of prescription are displayed in Table 3.

**TABLE 1:** Baseline characteristics of the included trials.

Study	Year	Sample size (Trial/control)	Average age (years)	Staging of DKD	Interventions	Duration (Week)	Report outcomes
Liao et al.	2019	60 (30/30)	58	NR	C: Valsartan	12	24 hUPRO
					T: Contrast + Jian pi li shi tong luo recipe		
Liu et al.	2019	60 (30/30)	58	NR	C: Irbesartan	3	24 hUPRO
					T: contrast + Xin liang huo xue recipe		
Du et al.	2018	80 (40/40)	60.02	NR	C: Valsartan	12	FBG, Scr, 24 hUPRO
					T: Contrast + Yi qi yang yin hua yu recipe		
Zeng et al.	2018	130 (65/65)	53.2	NR	C: Enalapril	24	FBG, HbA1C, TG
					Ta: Contrast + Nourishing yin, clearing heat and preventing prescription		
					Tb: Contrast + Jin gui Shen qi decoction		
He et al.	2017	100 (50/50)	52.6	NR	C: Perindopril	NR	FBG, Scr, TC, TG
					T: Contrast + Prescription for replenishing qi and nourishing yin, eliminating purge and dredging collaterals		
Fang et al.	2017	56 (28/28)	55.4	III	C: Fosinopril	12	BUN, FBG, HbA1C Scr, TC, TG
					T: Contrast + Prescription for replenishing qi, nourishing yin, strengthening kidney and invigorating spleen		
Su et al.	2017	120 (60/60)	60.42	III–IV	C: Irbesartan	8	Scr, TG, 24 hUPRO
					T: Contrast + Prescription for replenishing qi and nourishing yin and soothing the liver		
Shen et al.	2016	76 (38/38)	61.8	IV	C: Enalapril	12	FBG, HbA1C
					T: Contrast + Yi qi huo xue hua yu recipe		
Li et al.	2016	82 (41/41)	68.05	NR	C: ACEI/ARB	12	BUN, Scr
					T: Contrast + Yi qi wen yang hua yu recipe		
Bai et al.	2016	120 (60/60)	59.61	II–V	C: Irbesartan	12	FBG, TC, TG
					T: Contrast + Yi qi yang yin huo xue tong luo recipe		
Zhu et al.	2015	80 (40/40)	56.30	III–IV	C: Valsartan	8	BUN, Scr
					T: Contrast + Yi shen huo xue tong luo recipe		
Wu et al.	2015	98 (49/49)	58.6	III	C: Telmisartan	8	FBG, HbA1C, Scr
					T: Contrast + Yi qi hua yu recipe		
Peng et al.	2014	67 (32/32)	55.40	III–IV	C: Valsartan	8	BUN, Scr, 24 hUPRO
					T: Contrast + Jian pi yi shen tong luo recipe		
Li et al.	2015	144 (72/72)	56.12	NR	C: Enalapril		FBG, HbA1C, Scr 24 hUPRO
					T: Contrast + Bu shen huo xue recipe		
Fu et al.	2015	60 (30/30)	62.7	III–IV	C: Valsartan	52	FBG, HbA1C, Scr TC, 24 hUPRO
					T: Contrast + Yi qi bu shen		
					recipe + Tripterygium wilfordii		
Zhang et al.	2015	50 (25/25)	65.3	IV	C: Fosinopril	12	Scr, 24 hUPRO
					T: Contrast + Prescription for warming kidney and invigorating spleen		
Peng et al.	2015	151 (74/77)	56.5	III	C: Benazepril	12	FBG, HbA1C, TC, TG
					T: Zi shen huo xue recipe		
Yang et al.	2014	102 (60/42)	26–72	III–V	C: Benazepril	8	BUN, Scr
					T: Contrast + Yi shen huo xue decoction		
Wang et al.	2014	108 (54/54)	53.17	IV	C: Enalapril	4	FBG, Scr, 24 hUPRO
					T: Contrast + Zi shen qing re recipe		
Lu et al.	2014	60 (30/30)	64.1	IV	C: Irbesartan	12	BUN, Scr, HbA1C, 24 hUPRO
					T: Contrast + Zi shen qing re recipe		
Yang et al.	2013	80 (40/40)	55.8	III	C: Irbesartan	52	BUN, FBG, Scr, TG
					T: Contrast + Prescription for replenishing qi and nourishing yin, eliminating purge and dredging collaterals		
Sun et al.	2013	104 (52/52)	59.75	NR	C: Enalapril	8	FBG, HbA1C, 24 hUPRO
					T: Contrast + Bu shen huo xue recipe		
Ding et al.	2013	86 (45/41)	49.6	NR	C: Valsartan	NR	FBG, HbA1C, TC TG, 24 hUPRO
					T: Contrast + Qing re li shi huo xue recipe		
Deng et al.	2013	113 (56/57)	51.3	NR	C: Benazepril	8	FBG, TC, TG
					T: Contrast + Yi qi yang yin huo xue recipe		
Deng et al.	2013	60 (30/30)	52.5	NR	C: Irbesartan	12	BUN, HbA1C, Scr, FBG, TC, TG
					T: Contrast + Prescription for tonifying kidney and activating blood circulation		
Zhou et al.	2012	45 (24/21)	66.48	NR	C: Valsartan	12	FBG, HbA1C, TC Scr, TG
					T: Contrast + Bu shen huo xue qu feng recipe		
Zhao et al.	2012	100 (50/50)	68.2	III–IV	C: ACEI/ARB	12	BUN, FBG, Scr, HbA1C, 24 hUPRO
					T: Contrast + Yiqi yang yin tong luo huazhuo recipe		
Yan et al.	2012	60 (30/30)	48.1	NR	C: Basic treatment	12	FBG, HbA1C, Scr, 24 hUPRO
					T: Contrast + Bu shen yi qi huo xue recipe		
Wang et al.	2012	72 (36/36)	48.2	II	C: Enalapril	12	FBG, TC, TG
					T: Contrast + Jian pi bu shen huo xue hua tan recipe		
Wang et al.	2012	40 (20/20)	53.71	III	C: Benazepril	8	FBG, HbA1C, Scr, TC, TG
					T: Contrast + Bu shen yi qi huo xue recipe		
Guo et al.	2012	45 (24/21)	66.48	I–VI	C: Valsartan	12	FBG, HbA1C, TC, TG
					T: Contrast + Bu shen huo xue qu feng recipe		
Dou et al.	2015	100 (50/50)	48.6	NR	C: Enalapril	8	FBG, Scr, TC
					T: Contrast + Yi qi huo xue recipe		
Wu et al.	2011	96 (48/48)	45–76	NR	C: Benazepril	8	BUN, FBG, Scr, TC TG, 24 hUPRO
					T: Contrast + Prescription for tonifying qi and tonifying kidney and removing blood stasis		
Wang et al.	2011	60 (36/24)	40–76, average 66.8	NR	C: Irbesartan	NR	FBG, HbA1C
					T: Contrast + Bu shen yi qi huo xue recipe		
Yin et al.	2010	60 (30/30)	30.5 ± 13	NR	C: ACEI/ARB	NR	BUN, FBG, HbA1C, Scr
					T: contrast + Prescription for nourishing yin, replenishing qi, promoting dampness and dredging stasis		
Wang et al.	2010	75 (40/35)	50.2 ± 8.3	III	C: ACEI/ARB	NR	BUN, Scr, TC, TG
					T: Contrast + Yi qi bu shen recipe		
Wang et al.	2009	214 (107/107)	60.15–74.11	NR	C: Benazepril	12	BUN, FBG, Scr, TC, TG, 24 hUPRO
					T: Contrast + Yi shen pai zhuo recipe		
Feng et al.	2009	80 (42/38)	45 ± 8.0	IV	C: Benazepril	8	FBG, Scr, TC, TG
					T: Contrast + Nourishing kidney, activating blood circulation and promoting diuresis		
Fan et al.	2009	63 (31/32)	56.83 ± 11.6	NR	C: Basic treatment	8	BUN, Scr, TC, TG,24 hUPRO
					T: contrast + Tong mai oral liquid		
Chen et al.	2008	62 (31/31)	45–69, average 58.6	NR	C: Irbesartan	12	BUN, Scr, 24 hUPRO
					T: Replenishing qi and warming yang recipe		
Zhou et al.	2006	95 (48/47)	53.6 ± 6.2	III	C: Fosinopril/Valsartan	12	BUN, Scr, 24 hUPRO
					T: Qi di yi qi yang yin huoxue recipe		
Zhang et al.	2005	110 (60/50)	45–68, average 53.61	NR	C: Captopril	NR	BUN, FBG, HbA1C Scr, TC, TG
					T: Jiang tang yi shen decoction for invigorating the spleen and tonifying the kidney		
Wang et al.	2005	60 (40/20)	64.76 ± 9.75	NR	C: Captopril	8	BUN, FBG, HbA1C Scr, TC, TG, 24 hUPRO
					T: Method of tonifying spleen, tonifying kidney and activating blood circulation recipe		
Rui et al.	2002	46 (24/22)	38–70, average 55.5	NR	C: Enalapril	8–12	BUN, FBG, Scr, 24 hUPRO
					T: Prescription for tonifying kidney and tonifying qi, activating blood circulation and removing blood stasis recipe		

Abbreviations: BUN, blood urea nitrogen; DKD, diabetic kidney disease; FBG, fasting blood glucose; HbA1C, glycosylated hemoglobin; NR, not reported; Scr, serum creatinine; TC, total cholesterol; TG, triglyceride; 24hUPRO, 24-h urinary protein.

**TABLE 2:** Summary results of the outcome measures.

Main outcomes	No. studies	Sample size (Trial/control)	SMD	95% CI	*I* ^*2*^ (%)	*p*
BUN	19	1,568 (813/755)	−0.75	−1.10 to −0.40	91	0.114
FBG	20	1,673 (871/802)	−0.31	−0.47 to −0.15	60	0.615
HbA1C	20	1,673 (871/802)	−0.62	−0.89 to −0.36	85	0.277
Scr	19	1,568 (813/755)	−1.25	−1.69 to −0.81	94	0.783
TC	18	1,594 (829/765)	−0.95	−1.43 to −0.47	95	0.597
TG	18	1,594 (829/765)	−1.17	−1.76 to −0.59	96	0.738
24 h-UPRO	20	1,581 (805/776)	−1.10	−1.45 to −0.74	91	0.973

Abbreviations: BUN, blood urea nitrogen; CI, confidence interval; FBG, fasting blood glucose; HbA1C, glycosylated hemoglobin; *p,* egger’s test; Scr, serum creatinine; SMD, standardized mean difference; TC, total cholesterol; TG, triglyceride; 24 h-UPRO, 24-h urinary protein.

**TABLE 3:** Composition of traditional Chinese medicine reported in the included trials.

Study	Year	Prescription	Composition of drug
Liao et al.	2019	Jian-Pi-Li-Shi-Tong-Luo-Recipe	Astragali Radix (Huangqi), Atractylodis Macrocephalae Rhizoma (BaiZhu), Paeoniae Radix Alba (Baishao), Codonopsis Radix (Dangshen), Citri Reticulatae Pericarpium (Chenpi), Poria(Fuling), Salviae Miltiorrhizae Radix Et Rhizoma (Danshen), Chuanxiong Rhizoma (Chuanxiong), Alismatis Rhizoma (Zexie), Pheretima (Dilong), Liquidambaris Fructus (Lulutong), Hirudo (Shuizhi), Glycyrrhizae Radix Et Rhizoma (Gancao)
Liu et al.	2019	Xin-Liang-Huo-Xue-Recipe	Forsythiae Fructus (Lianqiao), Eucommiae Cortex (Duzhong), Lycii Fructus (Gouqizi), Lycopi Herba (Zelan), Glycyrrhizae Radix Et Rhizoma (Gancao), Paeoniae Radix Rubra (Chishao), Poria (Fuling)
Du et al.	2018	Yi-Qi-Yang-Yin-Hua-Yu-Recipe	Astragali Radix (Huangqi), Alismatis Rhizoma (Zexie), Rehmanniae Radix (Dihuang), Corni Fructus (Shanzhuyu), Poria (Fuling), Persicae Semen (Taoren), Carthami Flos (Honghua), Moutan Cortex (Mudanpi), Citri Reticulatae Pericarpium (Chenpi), Rhei Radix Et Rhizoma (Dahuang)
Zeng et al.	2018	Ta: Zi-Yin-Qing-Re-Recipe	Zi-Yin-Qing-Re-Recipe: Gypsum Fibrosum (Shigao), Glycyrrhizae Radix Et Rhizoma (Gancao), Glycyrrhizae Radix Et Rhizoma (Gancao), Ginseng Radix Et Rhizoma (Renshen), Coptidis Rhizoma (Huanglian), Phellodendri Chinensis Cortex (Huangbai), Anemarrhenae Rhizoma (Zhimu), Gardeniae Fructus (Zhizi), Poria (Fuling), Alismatis Rhizoma (Zexie), Angelicae Sinensis Radix (Danggui), Ophiopogonis Radix (Maidong), Forsythiae Fructus (Lianqiao), Armeniacae Semen Amarum (Kuxingren); Jin-Gui-shen-Qi-Recipe:Rehmanniae Radix Praeparata (Shudihuang), Dioscoreae Rhizoma (Shanyao), Corni Fructus (Shanzhuyu), Poria (Fuling), Moutan Cortex (Mudanpi), Calamus Lapidus (Shiwei), Poria (Fuling), Alismatis Rhizoma (Zexie), Cinnamomi Ramulus (Guizhi), Aconiti Lateralis Radix Praeparata (Fuzi), Achyranthis Bidentatae Radix (Niuxi), Plantaginis Semen (Cheqianzi), Lophatheri Herba (Danzhuye)
		Tb: Jin-Gui-shen-Qi- Recipe	
He et al.	2017	Yi-Qi-Yang-Yin-Xiao-Zheng-Tong-Luo- Recipe	Salviae Miltiorrhizae Radix Et Rhizoma (Danshen), Pheretima (Dilong), Astragali Radix (Huangqi), Poria (Fuling), Rhei Radix Et Rhizoma (Dahuang), Rehmanniae Radix (Dihuang), Centellae Herba (Jixuecao), Hirudo (Shuizhi), Amomi Fructus (Sharen), Trionycis Carapax (Biejia)
Fang et al.	2017	Yi-Qi-Yang-Yin-Gu-Shen-Jian-Pi- Recipe	Astragali Radix (Huangqi), Rehmanniae Radix (Dihuang), Dioscoreae Rhizoma (Shanyao), Rosae Laevigatae Fructus (Jinyingzi), Euryales Semen (Qianshi), Salviae Miltiorrhizae Radix Et Rhizoma (Danshen), Poria (Fuling), Epimedii Folium (Yiyanghuo), Hedyotis diffusa (Baihuashe shecao)
Su et al.	2017	Yi-Qi-Yang-Yin-Shu-Gan-Recipe	Astragali Radix (Huangqi), Adenophorae Radix (Nanshashen), Ophiopogonis Radix (Maidong), Poria (Fuling), Alismatis Rhizoma (Zexie), Dioscoreae Rhizoma (Shanyao), Corni Fructus (Shanzhuyu), Rehmanniae Radix (Dihuang), Eucommiae Cortex (Duzhong), Amomi Fructus (Sharen), Vinum Nightingale (Yejiaoteng), Paeoniae Radix Alba (Baishao), Cycyperi Rhizoma (Xiangfu), Glycyrrhizae Radix Et Rhizoma (Gancao)
Shen et al.	2016	Yi-Qi-Huo-Xue-Hua-Yu-Recipe	Astragali Radix (Huangqi), Salviae Miltiorrhizae Radix Et Rhizoma (Danshen), Chuanxiong Rhizoma (Chuanxiong), Corni Fructus (Shanzhuyu), Rehmanniae Radix (Dihuang), Pheretima (Dilong), Angelicae Sinensis Radix (Danggui), Dioscoreae Rhizoma (Shanyao), Glycyrrhizae Radix Et Rhizoma (Gancao), Polygonati Rhizoma (Huangjing), Hirudo (Shuizhi), Poria (Fuling),Atractylodis Rhizoma (Cangzhu)
Li et al.	2016	Yi-Qi-Wen-Yang-Hua-Yu-Recipe	Astragali Radix (Huangqi), Aconiti Lateralis Radix Praeparata (Fuzi), Salviae Miltiorrhizae Radix Et Rhizoma (Danshen), Paeoniae Radix Rubra (Chishao), Angelicae Sinensis Radix (Danggui), Hirudo (Shuizhi), Rosae Laevigatae Fructus (Jinyingzi), Euryales Semen (Qianshi), Epimedii Folium (Yinyanghuo), Cuscutae Semen (Tusizi), Poria (Fuling), Atractylodis Macrocephalae Rhizoma (BaiZhu), Notoginseng Radix Et Rhizoma (Sanqi), Alismatis Rhizoma (Zexie)
Bai et al.	2016	Yi-Qi-Yang-Yin-Huo-Xue-Tong-Luo -Recipe	Astragali Radix (Huangqi), Polygonati Rhizoma (Huangjing), Rehmanniae Radix (Dihuang), Salviae Miltiorrhizae Radix Et Rhizoma (Danshen), Pheretima (Dilong), Centellae Herba (Jixuecao), Rhei Radix Et Rhizoma (Dahuang)
Zhu et al.	2015	Yi-Shen-Huo-Xue-Tong-Luo-Recipe	Rehmanniae Radix Praeparata (Shudihuang), Corni Fructus (Shanzhuyu), Psoraleae Fructus (Buguzhi), Astragali Radix (Huangqi), Poria (Fuling), Dioscoreae Rhizoma (Shanyao), Lycii Fructus (Gouqizi), Euonymus Alatus (Guijianyu), Amomi Fructus (Sharen), Scutellariae Radix (Huanqin), Hirudo (Shuizhi), Glycyrrhizae Radix Et Rhizoma (Gancao)
Wu et al.	2015	Yi-Qi-Hua-Yu-Recipe	Astragali Radix (Huangqi), Scrophulariae Radix (Xuanshen), Angelicae Sinensis Radix (Danggui), Atractylodis Rhizoma (Cangzhu), Dioscoreae Rhizoma (Shanyao), Puerariae Lobatae Radix (Gegen), Salviae Miltiorrhizae Radix Et Rhizoma (Danshen), Pheretima (Dilong), Carthami Flos (Honghua), Rehmanniae Radix (Dihuang)
Peng et al.	2014	Jian-Pi-Yi-Shen-Tong-Luo-Recipe	Astragali Radix (Huangqi), Codonopsis Radix (Dangshen), Rehmanniae Radix Praeparata (Shudihuang), Poria (Fuling), Dioscoreae Rhizoma (Shanyao), Corni Fructus (Shanzhuyu), Cinnamomi Cortex (Rougui), Lycii Fructus (Gouqizi), Alismatis Rhizoma (Zexie), Euonymus Alatus (Guijianyu), Amomi Fructus (Sharen), Psoraleae Fructus (Buguzhi), Taraxaci Herba (Pugongying), Hirudo (Shuizhi), Glycyrrhizae Radix Et Rhizoma (Gancao)
Li et al.	2015	Bu-Shen-Huo-Xue-Recipe	Astragali Radix (Huangqi), Rehmanniae Radix (Dihuang), Rubi Fructus (Fupenzi), Smilacis Glabrae Rhizoma (Tufuling), Salviae Miltiorrhizae Radix Et Rhizoma (Danshen), Chuanxiong Rhizoma (Chuanxiong), Paeoniae Radix Rubra (Chishao), Eucommiae Cortex (Duzhong), Sparganii Rhizoma (Sanleng), Curcumae Rhizoma (Ezhu), Schisandrae Chinensis Fructus (Wuweizi), Plantaginis Herba (Cheqiancao)
Fu et al.	2015	Yi-Qi-Bu-Shen-Recipe	Astragali Radix (Huangqi), Codonopsis Radix (Dangshen), Salviae Miltiorrhizae Radix Et Rhizoma (Danshen), Angelicae Sinensis Radix (Danggui), Poria (Fuling), Dioscoreae Rhizoma (Shanyao), Spatholobi Caulis (Jixueteng), Corni Fructus (Shanzhuyu), Rosae Laevigatae Fructus (Jinyingzi), Curcumae Rhizoma (Ezhu)
Zhang et al.	2015	Wen-Shen-Jian-Pi-Recipe	Curculiginis Rhizoma (Xianmao), Epimedii Folium (Yiyanghuo), Euryales Semen (Qianshi), Rosae Laevigatae Fructus (Jinyingzi), Astragali Radix (Huangqi), Atractylodis Macrocephalae Rhizoma (BaiZhu), Poria (Fuling), Rhei Radix Et Rhizoma (Dahuang), Salviae Miltiorrhizae Radix Et Rhizoma (Danshen), Chuanxiong Rhizoma (Chuanxiong), Curcumae Rhizoma (Ezhu)
Peng et al.	2015	Zi-Shen-Huo-Xue-Recipe	Astragali Radix (Huangqi), Rehmanniae Radix (Dihuang), Dioscoreae Rhizoma (Shanyao), Corni Fructus (Shanzhuyu), Houttuyniae Herba (Yuxingcao), Salviae Miltiorrhizae Radix Et Rhizoma (Danshen), Rhei Radix Et Rhizoma (Dahuang), Chuanxiong Rhizoma (Chuanxiong), Hirudo (Shuizhi), Euonymus Alatus (Guijianyu), 汉Stephaniae Tetrandrae Radix (Fangji), Epimedii Folium (Yinyanghuo), Ligustri Lucidi Fructus (Nvzhenzi), Cuscutae Semen (Tusizi), Glycyrrhizae Radix Et Rhizoma (Gancao)
Yang et al.	2014	Yi-Shen-Huo-Xue- Recipe	Astragali Radix (Huangqi), Polygonati Rhizoma (Huangjing), Salviae Miltiorrhizae Radix Et Rhizoma (Danshen), Rehmanniae Radix Praeparata (Shudihuang), Puerariae Lobatae Radix (Gegen), Dioscoreae Rhizoma (Shanyao), Corni Fructus (Shanzhuyu), Poria (Fuling), Alismatis Rhizoma (Zexie), Moutan Cortex (Mudanpi), Hirudo (Shuizhi)
Wang et al.	2014	Zi-Shen-Qing-Re-Recipe	Astragali Radix (Huangqi), Polygonati Rhizoma (Huangjing), Salviae Miltiorrhizae Radix Et Rhizoma (Danshen), Rehmanniae Radix Praeparata (Shudihuang), Puerariae Lobatae Radix (Gegen), Dioscoreae Rhizoma (Shanyao), Corni Fructus (Shanzhuyu), Poria (Fuling), Alismatis Rhizoma (Zexie), Moutan Cortex (Mudanpi), Hirudo (Shuizhi)
Lu et al.	2014	Yi-Shen-Recipe	Rehmanniae Radix Praeparata (Shudihuang), Morindae Officinalis Radix (Bajitian), Dioscoreae Rhizoma (Shanyao), Corni Fructus (Shanzhuyu), Angelicae Sinensis Radix (Danggui), Paeoniae Radix Rubra (Chishao), Aconiti Lateralis Radix Praeparata (Fuzi), Epimedii Folium (Yinyanghuo), Zingiberis Rhizoma (Ganjiang)
Yang et al.	2013	Yi-Qi-Yang-Yin-Xiao-Zheng-Tong-Luo- Recipe	Astragali Radix (Huangqi), Salviae Miltiorrhizae Radix Et Rhizoma (Danshen), Centellae Herba (Jixuecao), Poria (Fuling), Pheretima (Dilong), Rehmanniae Radix (Dihuang), Amomi Fructus (Sharen), Trionycis Carapax (Biejia), Rhei Radix Et Rhizoma (Dahuang)
Sun et al.	2013	Bu-Shen-Huo-Xue-Recipe	Astragali Radix (Huangqi), Panica Pastoris Flos (Jicaihua), Cassiae Semen (Juemingzi), Rhei Radix Et Rhizoma (Dahuang), Rubi Fructus (Fupenzi), Smilacis Glabrae Rhizoma (Tufuling), Plantaginis Semen (Cheqianzi), Plantaginis Herba (Cheqiancao), Salviae Miltiorrhizae Radix Et Rhizoma (Danshen), Chuanxiong Rhizoma (Chuanxiong), Paeoniae Radix Rubra (Chishao), Schisandrae Chinensis Fructus (Wuweizi), Eucommiae Cortex (Duzhong), Rehmanniae Radix (Dihuang), Sparganii Rhizoma (Sanleng), Curcumae Rhizoma (Ezhu), Scutellariae Barbatae Herba (Banzhilian)
Ding et al.	2013	Qing-Re-Li-Shi-Huo-Xue-Recipe	Hedyotis diffusa (Baihuasheshecao), Plantaginis Herba (Cheqiancao), Calamus Lapidus (Shiwei), Lycopi Herba (Zelan), Salviae Miltiorrhizae Radix Et Rhizoma (Danshen), Spatholobi Caulis (Jixueteng), Rehmanniae Radix (Dihuang), Dioscoreae Rhizoma (Shanyao), Coicis Semen (Yiyiren), Poria (Fuling), Cicadae Periostracum (Chantui), Bombyx Batryticatus (Jiangcan), Aurantii Fructus (Zhike)
Deng et al.	2013	Yi-Qi-Yang-Yin-Huo-Xue-Recipe	Astragali Radix (Huangqi), Pseudostellariae Radix (Taizishen), Rehmanniae Radix (Dihuang), Dioscoreae Rhizoma (Shanyao), Corni Fructus (Shanzhuyu), Moutan Cortex (Mudanpi), Poria (Fuling), Alismatis Rhizoma (Zexie), Chuanxiong Rhizoma (Chuanxiong), Notoginseng Radix Et Rhizoma (Sanqi)
Deng et al.	2013	Bu-Shen-Huo-Xue-Recipe	Draconis Sanguis (Xuejie), Notoginseng Radix Et Rhizoma (Sanqi), Pseudostellariae Radix (Taizishen), Dendrobii Caulis (Shihu), Lycii Fructus (Gouqizi), Salviae Miltiorrhizae Radix Et Rhizoma (Danshen)
Zhou et al.	2012	Bu-Shen-Huo-Xue-Qu-Feng-Recipe	Astragali Radix (Huangqi), Psoraleae Fructus (Buguzhi), Salviae Miltiorrhizae Radix Et Rhizoma (Danshen), Hirudo (Shuizhi), Pinelliae Rhizoma (Banxia), Arisaema Cum Bile (Dannanxing), Sinomenii Caulis (Qingfengteng)
Zhao et al.	2012	Yi-Qi-Yang-Yin-Tong-Luo-Hua-Zhuo- Recipe	Astragali Radix (Huangqi), Rehmanniae Radix (Dihuang), Corni Fructus (Shanzhuyu), Dioscoreae Rhizoma (Shanyao), Polygonati Rhizoma (Huangjing), Atractylodis Rhizoma (Cangzhu), Citri Reticulatae Pericarpium (Chenpi), Salviae Miltiorrhizae Radix Et Rhizoma (Danshen), Lycopi Herba (Zelan), Persicae Semen (Taoren)
Yan et al.	2012	Bu-Shen-Yi-Qi-Huo-Xue-Recipe	Astragali Radix (Huangqi), Codonopsis Radix (Dangshen), Polygonati Rhizoma (Huangjing), Poria (Fuling), Corni Fructus (Shanzhuyu), Rehmanniae Radix (Dihuang), Ophiopogonis Radix (Maidong), Schisandrae Chinensis Fructus (Wuweizi), Alismatis Rhizoma (Zexie), Eucommiae Cortex (Duzhong), Achyranthis Bidentatae Radix (Niuxi), Polygoni Cuspidati Rhizoma Et Radix (Huzhang), Hirudo (Shuizhi), Moutan Cortex (Mudanpi), Salviae Miltiorrhizae Radix Et Rhizoma (Danshen), Glycyrrhizae Radix Et Rhizoma (Gancao)
Wang et al.	2012	Jian-Pi-Bu-Shen-Huo-Xue-Hua-Tan- Recipe	Astragali Radix (Huangqi), Poria (Fuling), Atractylodis Macrocephalae Rhizoma (BaiZhu), Atractylodis Rhizoma (Cangzhu), Scrophulariae Radix (Xuanshen), Corni Fructus (Shanzhuyu), Rehmanniae Radix Praeparata (Shudihuang), Dioscoreae Rhizoma (Shanyao), Salviae Miltiorrhizae Radix Et Rhizoma (Danshen), Citri Reticulatae Pericarpium (Chenpi), Glycyrrhizae Radix Et Rhizoma (Gancao)
Wang et al.	2012	Bu-Shen-Yi-Qi-Huo-Xue-Recipe	Astragali Radix (Huangqi), Dioscoreae Rhizoma (Shanyao), Atractylodis Macrocephalae Rhizoma (BaiZhu), Rehmanniae Radix Praeparata (Shudihuang), Mori Fructus (Sangshen), Corni Fructus (Shanzhuyu), Chuanxiong Rhizoma (Chuanxiong), Notoginseng Radix Et Rhizoma (Sanqi), Salviae Miltiorrhizae Radix Et Rhizoma (Danshen)
Guo et al.	2012	Bu-Shen-Huo-Xue-Qu-Feng-Recipe	Astragali Radix (Huangqi), Psoraleae Fructus (Buguzhi), Salviae Miltiorrhizae Radix Et Rhizoma (Danshen), Hirudo (Shuizhi), Pinelliae Rhizoma (Banxia), Arisaema Cum Bile (Dannanxing), Sinomenii Caulis (Qingfengteng)
Dou et al.	2015	Yi-Qi-Huo-Xue-Recipe	Astragali Radix (Huangqi), Salviae Miltiorrhizae Radix Et Rhizoma (Danshen), Rhei Radix Et Rhizoma (Dahuang), Chuanxiong Rhizoma (Chuanxiong), Dioscoreae Rhizoma (Shanyao), Trichosanthis Radix (Tianhuafen), Atractylodis Rhizoma (Cangzhu), Schisandrae Chinensis Fructus (Wuweizi)
Wu et al.	2011	Yi-Qi-Bu-Shen-Qu-Yu-Recipe	Testudinis Carapax Et Plastrum (Guijia), Trionycis Carapax (Biejia), Pseudostellariae Radix (Taizishen), Poria (Fuling), Dioscoreae Rhizoma (Shanyao), Polygonati Rhizoma (Huangjing), Salviae Miltiorrhizae Radix Et Rhizoma (Danshen), Notoginseng Radix Et Rhizoma (Sanqi)
Wang et al.	2011	Bu-Shen-Yi-Qi-Huo-Xue-Recipe	Astragali Radix (Huangqi), Angelicae Sinensis Radix (Danggui), Rehmanniae Radix Praeparata (Shudihuang), Corni Fructus (Shanzhuyu), Dioscoreae Rhizoma (Shanyao), Alismatis Rhizoma (Zexie), Poria (Fuling), Moutan Cortex (Mudanpi), Salviae Miltiorrhizae Radix Et Rhizoma (Danshen), Mantidis Oötheca (Sangpiaoxiao)
Yin et al.	2010	Yang-Yin-Yi-Qi-Li-Shi-Tong-Yu-Recipe	Rehmanniae Radix (Dihuang), Adenophorae Radix (Nanshashen), Glehniae Radix (Beishashen), Asparagi Radix (Tiandong), Ophiopogonis Radix (Maidong), Pseudostellariae Radix (Taizishen), Astragali Radix (Huangqi), Salviae Miltiorrhizae Radix Et Rhizoma (Danshen), Moutan Cortex (Mudanpi), Corn Whisker (Yumixu), Coicis Semen (Yiyiren), Euonymus Alatus (Guijianyu), Feles Herbam Ferret (Maozhuacao), Imperatae Rhizoma (Baimaogen)
Wang et al.	2010	Yi-Qi-Bu-Shen-Recipe	Astragali Radix (Huangqi), Polygoni Multiflori Caulis (Shouwuteng), Lycii Fructus (Gouqizi), Polygonati Rhizoma (Huangjing), Pseudostellariae Radix (Taizishen), Corni Fructus (Shanzhuyu), Rehmanniae Radix (Dihuang), Salviae Miltiorrhizae Radix Et Rhizoma (Danshen), Rosae Laevigatae Fructus (Jinyingzi), Citri Reticulatae Pericarpium (Chenpi), Paeoniae Radix Alba (Baishao), Leonuri Herba (Yimucao), Rhei Radix Et Rhizoma (Dahuang), Dioscoreae Rhizoma (Shanyao), Angelicae Sinensis Radix (Danggui), Moutan Cortex (Mudanpi)
Wang et al.	2009	Yi-Shen-Pai-Zhuo-Recipe	Rhei Radix Et Rhizoma (Dahuang), Astragali Radix (Huangqi), Poria (Fuling), Alismatis Rhizoma (Zexie), Salviae Miltiorrhizae Radix Et Rhizoma (Danshen), Paeoniae Radix Rubra (Chishao), Ephedrae Herba (Mahuang), Pinelliae Rhizoma (Banxia)
Feng et al.	2009	Bu-Shen-Huo-Xue-Li-Shui-Recipe	Astragali Radix (Huangqi), Codonopsis Radix (Dangshen), Corni Fructus (Shanzhuyu), Ligustri Lucidi Fructus (Nvzhenzi), Dioscoreae Rhizoma (Shanyao), Poria (Fuling), Atractylodis Macrocephalae Rhizoma (BaiZhu), Alismatis Rhizoma (Zexie), Salviae Miltiorrhizae Radix Et Rhizoma (Danshen), Puerariae Lobatae Radix (Gegen), Angelicae Pubescentis Radix (Duhuo), Chuanxiong Rhizoma (Chuanxiong)
Fan et al.	2009	Tong-Mai-Oral liquid	Puerariae Lobatae Radix (Gegen), Salviae Miltiorrhizae Radix Et Rhizoma (Danshen), Chuanxiong Rhizoma (Chuanxiong)
Chen et al.	2008	Yi-Qi-Wen-Yang-Recipe	Astragali Radix (Huangqi), Ginseng Radix Et Rhizoma (Renshen), Cinnamomi Ramulus (Guizhi), Atractylodis Macrocephalae Rhizoma (BaiZhu), Cimicifugae Rhizoma (Shengma), Bupleuri Radix (Chaihu), Angelicae Sinensis Radix (Danggui), Glycyrrhizae Radix Et Rhizoma (Gancao), Persicae Semen (Taoren), Chuanxiong Rhizoma (Chuanxiong), Paeoniae Radix Rubra (Chishao), Rehmanniae Radix (Dihuang), Aurantii Fructus (Zhike), Achyranthis Bidentatae Radix (Niuxi), Platycodonis Radix (Jiegeng), Pheretima (Dilong)
Zhou et al.	2006	Qi-Di-Yi-Qi-Yang-Yin-Huo-Xue- Recipe	Astragali Radix (Huangqi), Rehmanniae Radix (Dihuang), Puerariae Lobatae Radix (Gegen), Dioscoreae Rhizoma (Shanyao), Leonuri Herba (Yimucao), Salviae Miltiorrhizae Radix Et Rhizoma (Danshen), Chuanxiong Rhizoma (Chuanxiong), Rhei Radix Et Rhizoma (Dahuang), Poria (Fuling), Alismatis Rhizoma (Zexie), Glycyrrhizae Radix Et Rhizoma (Gancao)
Zhang et al.	2005	Jian-Pi-Bu-Shen-Jiang-Tang-Yi-Shen- Recipe	Rehmanniae Radix (Dihuang), Rehmanniae Radix Praeparata (Shudihuang), Astragali Radix (Huangqi), Pseudostellariae Radix (Taizishen), Salviae Miltiorrhizae Radix Et Rhizoma (Danshen), Poria (Fuling), Dioscoreae Rhizoma (Shanyao), Corni Fructus (Shanzhuyu), Lycii Fructus (Gouqizi), Cuscutae Semen (Tusizi), Polyporus (Zhuling), Alismatis Rhizoma (Zexie), Aucklandiae Radix (Muxiang), Amomi Fructus (Sharen), Chaenomelis Fructus (Mugua), Plantaginis Semen (Cheqianzi)
Wang et al.	2005	Bu-Pi-Yu-Shen-Huo-Xue-Recipe	Astragali Radix (Huangqi), Codonopsis Radix (Dangshen), Rehmanniae Radix Praeparata (Shudihuang), Dioscoreae Rhizoma (Shanyao), Corni Fructus (Shanzhuyu), Poria (Fuling), Moutan Cortex (Mudanpi), Alismatis Rhizoma (Zexie), Atractylodis Macrocephalae Rhizoma (BaiZhu), Magnoliae Officinalis Cortex (Houpo), Amomi Fructus (Sharen), Cuscutae Semen (Tusizi), Lycii Fructus (Gouqizi), Salviae Miltiorrhizae Radix Et Rhizoma (Danshen), Persicae Semen (Taoren), Carthami Flos (Honghua), Leonuri Herba (Yimucao), Rhei Radix Et Rhizoma (Dahuang)
Rui et al.	2002	Bu-Shen-Yi-Qi-Huo-Xue-Hua-Yu-Recipe	Astragali Radix (Huangqi), Aconiti Lateralis Radix Praeparata (Fuzi), Rehmanniae Radix Praeparata (Shudihuang), Corni Fructus (Shanzhuyu), Cinnamomi Ramulus (Guizhi), Alismatis Rhizoma (Zexie), Poria (Fuling), Leonuri Herba (Yimucao), Dioscoreae Rhizoma (Shanyao), Chuanxiong Rhizoma (Chuanxiong), Salviae Miltiorrhizae Radix Et Rhizoma (Danshen)

### 3.3 Quality assessment (Risk of bias)

A summary of the risk of bias of the 44 RCTs is shown in Supplementary Figure S1. Two of the seven domains (allocation concealment and blinding methods) based on the Cochrane tool were rated as high risk of bias. Other limitations were identified as unclear risk of random sequence generation (54.5%), binding of outcome assessment (97.7%), and selective reporting (68.2%).

### 3.4 Outcomes

#### 3.4.1 Primary outcome

##### 3.4.1.1 24-h UPRO

**FIGURE 2:**
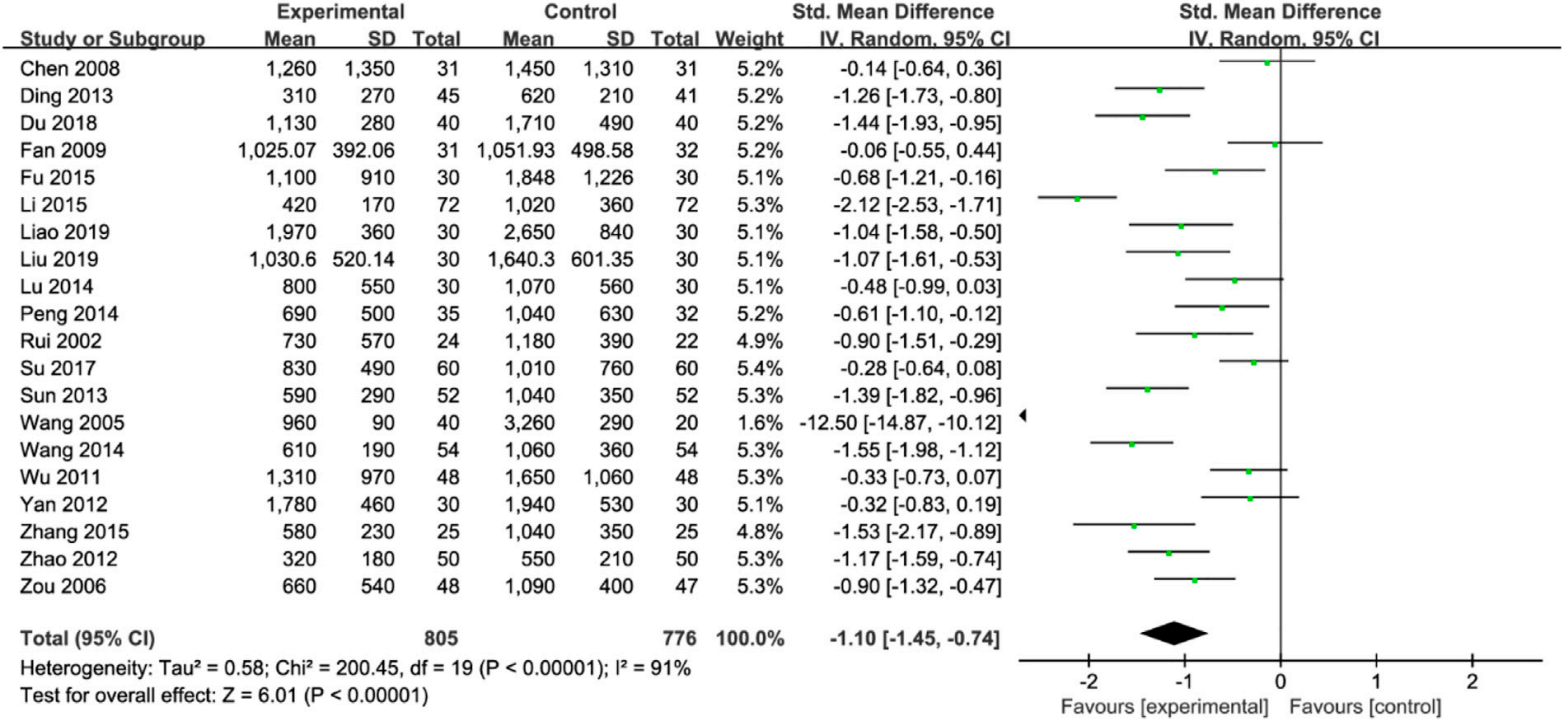
Forest plot for meta-analysis of the effect of TCM combined with Western medicine on 24-h urinary protein.

A summary of the effect estimate of the primary outcome is presented in Figure 2. In our analysis, TCM combined with Western medicine showed a greater improvement in the reduction of 24-h UPRO than Western medicine alone, with an SMD of −1.10 [95% confidence interval (CI) −1.45 to −0.74; *n* = 20 studies].

**FIGURE 3:**
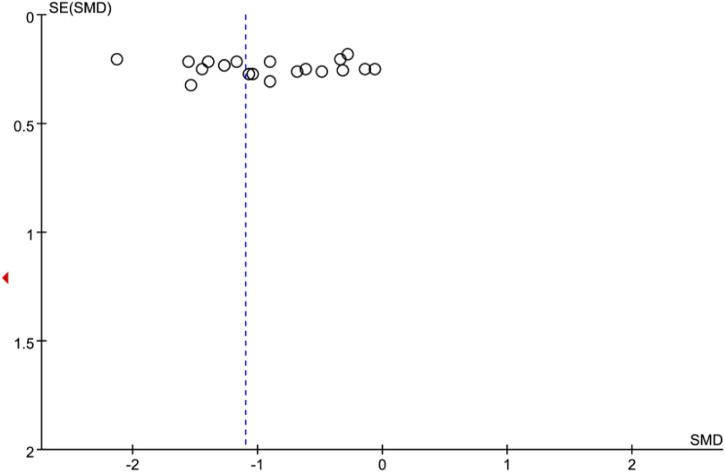
Funnel plot for meta-analysis of the effect of TCM combined with Western medicine on 24-h urinary protein.

However, high interstudy heterogeneity was noted (I^2^ = 91%). We tried to investigate the potential sources of heterogeneity through subgroup analyses stratified by Western medicine intervention measures, study sample size, year of publication, treatment duration, duration of DKD, mean patient age and staging of DKD. The effect estimates of all the subgroups were consistent with the primary effect estimate, indicating that the result of the meta-analysis of the primary outcome was robust. Moreover, the I^2^ values for most of the subgroups were slightly or moderately reduced (Table 4), inferring that the stratified factors might be potential sources of heterogeneity. No publication bias was noted from the general inspection of funnel plot symmetry (Figure 3) and Egger’s test (*p* = 0.973).

**TABLE 4:** Subgroup analyses for the effect of 24-h urinary protein.

Variables	No. Studies	Trial no.	Control no.	SMD	95%CI	*I* ^2^ (%)	*p* for interaction
Western medicine treatment							0.05
ACEI	13	574	552	−1.35	−1.88 to −0.83	93	
ARB	7	231	224	−0.75	−1.05 to −0.46	58	
Sample size							0.97
≤70	11	336	312	−1.14	−1.74 to −0.55	92	
>70	9	469	464	−1.51	−1.56 to −0.75	88	
Year of publication							0.13
Before 2010	5	174	152	−2.1	−3.47 to −0.73	96	
Year 2010 and after	14	601	594	−1.01	−1.33 to −0.70	85	
Duration of treatment							0.14
≤8 weeks	8	350	328	−1.46	−0.22 to −0.69	95	
>8 weeks	10	338	335	−0.85	−1.13 to −0.57	66	
Duration of DKD							0.03
<10 years	4	152	129	−3.17	−4.90 to −1.44	97	
≥10 years	4	186	186	−1.12	−1.62 to −0.63	80	
Average age							0.06
<60 years	15	605	596	−0.96	−1.27 to −0.65	84	
≥60 years	5	200	180	−2.23	−3.52 to −0.93	96	
Staging of DKD							0.32
Staging III	2	83	79	−0.78	−1.10 to −0.45	0	
Staging III-IV	3	152	152	−1.33	−2.16 to −0.51	90	
Staging IV	3	109	109	−1.18	−1.89 to −0.47	82	

Abbreviations: ACEI, angiotensin-converting enzyme inhibitor; ARB, angiotensin receptor blocker; CI, confidence interval; DKD, diabetes kidney disease; SMD, standardized mean difference.

#### 3.4.2 Secondary outcomes

##### 3.4.2.1 Fasting blood glucose

FBG levels were reported by 20 studies, with 871 participants in the interventional (TCM combined with Western medicine) group and 802 participants in the control (Western medicine alone) group. TCM combined with Western medicine significantly reduced the FBG level more than Western medicine alone [SMD: −0.31 (95% CI: −0.47 to −0.15)]. Subgroup analysis indicated that compared with that in patients treated with the control intervention, the FBG level was more significantly reduced in those with a longer course of treatment (>8 weeks) [SMD: −0.45 (95% CI: −0.68 to −0.22)] than in those with a shorter course of treatment (≤8 weeks) and in those with an older average age (≥60 years) [SMD −0.34 (95% CI: −0.54 to −0.14)] than in those with a younger average age (<60 years) (Supplementary Table S1).

##### 3.4.2.2 Glycosylated hemoglobin

The HbA1c level was reported by 20 studies with 871 participants in the interventional group and 802 participants in the control group. Overall, the HbA1c level was significantly lower in the interventional group than in the control group [SMD: −0.62 (95% CI, −0.89 to −0.36)]. In the subgroup analysis, HbA1c levels were significantly lower in the interventional group among different subgroups stratified by baseline characteristics (Supplementary Table S2). However, there was no significant difference between the subgroups.

##### 3.4.2.3 Serum creatinine

The Scr level was reported by 19 studies with 813 participants in the interventional group and 755 participants in the control group. Compared to the control group, the interventional group had a significantly lower Scr level [SMD −1.25 (95% CI: −1.69 to −0.81)]. In the subgroup analysis, the Scr level was significantly lower in those with a shorter duration of DKD (≤10 years) [SMD −2.81 (95% CI: −4.08 to −1.54)] than in those with a longer duration of DKD (>10 years) and in those with a younger average age (≤70 years) [SMD −0.92 (95% CI: −1.61 to −0.23)] than in those with an older average age (>70 years) (Supplementary Table S3).

##### 3.4.2.4 Blood urea nitrogen

The BUN level was reported by four studies with 813 participants in the investigational group and 755 participants in the control group. The investigational group had a significantly higher BUN level than the control group [SMD −0.75 (95% CI: −1.10, −0.40)]. In the subgroup analysis, the BUN level was significantly lower in those with a shorter duration of DKD (<10 years) [SMD −1.30 (95% CI: −2.15 to −0.46)] than in those with a longer duration of DKD (≥10 years) and in those with an older average age (≥60 years) [SMD −1.44 (95% CI: −2.47 to −0.41)] than in those with a younger average age (<60 years) (Supplementary Table S4).

##### 3.4.2.5 Total cholesterol

The TC level was reported by 18 studies with 829 participants in the interventional group and 765 participants in the control group. Compared to the control group, the interventional group had significantly lower TC levels [SMD −0.95 (95% CI: −1.43 to −0.47)]. In the subgroup analysis, the TC level was significantly lower in those with a longer duration of DKD (>5 years) [SMD −1.79 (95% CI: −2.57 to −1.02)] than in those with a shorter duration of DKD (≤5 years) and in those with an older average age (≥60 years) [SMD −1.36 (95% CI: −3.37 to −0.64)] than in those with a younger average age (<60 years) (Supplementary Table S5).

##### 3.4.2.6 Triglyceride

TG was reported by 18 studies with 829 participants in the interventional group and 765 participants in the control group. Compared to the control group, the interventional group had a significantly lower TG level [SMD −1.17 (95% CI: −1.76 to −0.59)]. Subgroup analysis showed that compared with the control group, for the interventional group, the TG level was significantly lower for trials with larger sample sizes (>70) [SMD −1.41 (95% CI: −2.17 to −0.64)] than for trials with smaller sample sizes (*p* < 0.001) (Supplementary Table S6).

## 4 Discussion

### 4.1 Principal findings

This meta-analysis found that compared to Western medicine alone, traditional Chinese medicine (TCM) combined with Western medicine yielded significantly better clinical efficacy in the treatment of proteinuria in patients with diabetic kidney disease (DKD).

The 24-h urinary protein (24-h UPRO) level was significantly more improved in the intervention group than in the control group. Moreover, the levels of fasting blood glucose (FBG), glycosylated hemoglobin (HbA1c), blood urea nitrogen (BUN), total cholesterol (TC) and triglyceride (TG) were also generally more improved with the intervention of TCM combined with Western medicine.

### 4.2 Potential mechanisms

The potential mechanisms of TCM in the treatment of DKD and albuminuria are not clear. In recent years, under the guidance of the unique theoretical system of TCM, we found in clinical practice that TCM could alleviate the clinical symptoms related to DKD and improve renal function. Animal experiments have shown that astragaloside IV can relieve proteinuria and glomerulosclerosis in streptozotocin (STZ)-induced DKD mice, inhibit podocyte apoptosis, restore damaged autophagy, block autophagy or AMPK activation, and block the effect of astragaloside IV, suggesting that astragaloside IV partially delays the progression of DKD through AMPK-mediated autophagy induction (Du et al., 2018). Mangiferin is a natural xanthone extracted from Anemarrhena and other plants, and recent studies have shown that mangiferin can delay the progression of DKD in STZ-induced DKD rats and protect podocytes (Wang et al., 2018). Berberine is an extract of Coptis chinensis and Phellodendron Phellodendri that has the pharmacological effects such as reducing blood glucose and lipid levels and anti-inflammatory effects (Jin F. et al., 2017; Jin Y. et al., 2017; Sun et al., 2018). Adzuki bean extract has been reported to reduce the level of plasma glutathione and block the expression of heme oxygenase superoxide dismutase 1 and p47phox protein in DKD rats, which is consistent with the improvement of renal dysfunction and glucose metabolism disorder (Sato et al., 2016). Abelmoschus Manihot is an extract of okra that shows a nephroprotective effect by improving podocytosis and alleviating renal pathological changes in type 2 diabetic rats (Kim et al., 2018). It has received increasing attention in the treatment of DKD.

In the treatment of CKD with Western medicine, some commonly used drugs (such as hormones and immunosuppressants) are toxic, which may cause severe side effects and can affect patient quality of life during the treatment. The application of TCM combined with Western medicine according to the syndrome differentiation has been proved to not only increase the curative effect, prevent the rebound phenomenon, but also reduce the side effects such as Cushing syndrome, mental symptoms, and infection.

### 4.3 Implications

This meta-analysis found that compared to Western medicine alone, TCM combined with Western medicine has significant effects on reducing 24-h UPRO and improves renal function indices and lipid profiles compared with Western medicine alone for DKD. The results of the study will provide clinicians with the best choice for the treatment of DKD proteinuria and provide them with a research direction. The use of TCM combined with Western medicine in the treatment of DKD may improve the therapeutic effect.

### 4.4 Comparisons with previous reports

Some of the findings of this meta-analysis are in line with the findings of a previously published meta-analysis, which mainly focused on the effects of single herbs or specific formulations. Ren et al. (2019) investigated the clinical efficacy of Tripterygium wilfordii polyglycosides in the treatment of stage IV DKD and found that Tripterygium wilfordii polyglycosides could induce a significant decrease in albuminuria and Scr and increase in albumin. Based on 21 RCTs, it was found that Tripterygium wilfordii polyglycosides combined with ARB was superior to ACEI in reducing 24-h proteinuria. A recently published article regarding the Liuwei Dihuang Pill in the treatment of proteinuria in DKD showed that compared with TCM placebo, Liuwei Dihuang Pill had a better clinical effect in patients with DKD, but there was no significant difference in the HbA1c level (Ren et al., 2019). Though the results indicated that the effects of FBG and HbA1c examined in the study had great heterogeneity, the data suggested that the interventions of different prescriptions might be the reason for the heterogeneity, suggesting that different prescriptions had different effects on patients with DKD.

### 4.5 Strengths and limitations

Our study has several strengths. Firstly, this is the largest and most comprehensive pooled analysis regarding this topic which may provide high level evidence on the efficacy of TCM combined with Western medicine in the treatment of DKD. Secondly, we enumerated the compositions of each prescription for each trial so that the between-prescription differences could be more transparent (Table 4). Thirdly, compared with single prescriptions of TCM, this study included all the prescriptions of TCM for the treatment of DKD under the guidance of different principles of TCM treatment, which provides a more objective and comprehensive evaluation of TCM combined with Western medicine in the treatment of proteinuria in DKD patients. In addition, it is well known that instead of analyzing a single component of TCM, since each TCM contains a variety of compounds, it is better to analyze the whole TCM; for example, *Astragalus membranaceus* contains triterpene saponins, flavonoids, polysaccharides, and other components (Liu et al., 2020; Salehi et al., 2020; Guo et al., 2021). Thus, this study conducted a more comprehensive evaluation of the effects of TCM prescriptions at the multicomponent, multitarget and multipathway levels, which provided broadened new ideas for the treatment of proteinuria in DKD patients. Finally, the current systematic review involved the six most frequently assessed outcome indicators with the largest combined sample size. We also conducted multiple subgroup analyses to investigate the sources of heterogeneity and the robustness of the findings on the therapeutic effect of TCM on proteinuria and other serum indicators, providing high-level evidence for TCM combined with Western medicine in the treatment of DKD.

However, our study still has several limitations. Firstly, several trials included in our meta-analysis had a relatively small sample size, making some subgroup analyses less robust. Secondly, 17 of the included trials had a short follow-up period (<12 weeks), and the long-term effects of TCM on renal function and clinical outcomes should be further investigated in the future. Thirdly, more than half of the included trials did not provide details of the randomization and allocation procedures, so the impact of potential selection bias is unclear. Fourthly, since most of the participants in the trials were middle-aged and elderly individuals, the renal protective effect of TCM on young people and those with advanced kidney disease is still uncertain. In the included trials, the form of TCM used was a multicomponent TCM prescription developed according to the clinical experience with classic prescriptions or of famous TCM experts. At present, the drug-drug interactions and the detailed components of these TCM prescriptions are not clear. Fifthly, we did not test the side effects of TCM, which will be further focused on in our future studies. Finally, though we investigated the potential sources of heterogeneity through multiple subgroup analyses, the heterogeneity remained high. The difference in TCM composition could be one of the sources of heterogeneity.

This study has potential risk of bias in several aspects. Firstly, according to the risk of bias assessment of the included trials, we found that most of the trials had an obvious risk of bias in allocation concealment and blinding methods. Secondly, due to the lack of sufficient data, we only evaluated the overall clinical efficacy of TCM in the treatment of DKD, while not the effect of specific chemical components of TCM on DKD. Finally, the difference in observation time between trials might also influence the results. However, the results of multiple subgroup analyses and sensitivity analysis confirmed the robustness of the pooled estimates.

## 5 Conclusion

Based on this systematic review and meta-analysis of large sample size RCTs, we found that TCM combined with Western medicine has significant effects on reducing 24-h UPRO and improves renal function indices and lipid profiles compared with Western medicine alone for DKD. However, the results should be interpreted with caution due to the high heterogeneity and risk of bias of the included trials. This study also provides a theoretical basis for potential prescription selection and dispensing for further research. In the future, we propose that larger, well-designed, multicenter RCTs with long-term follow-up should be carried out to further confirm the long-term efficacy and safety of TCM in the treatment of DKD.

## Data Availability

The original contributions presented in the study are included in the article/Supplementary Material, further inquiries can be directed to the corresponding authors.

## Abbreviations

ACEI, angiotensin-converting–enzyme inhibitor; ARB, angiotensin II receptor blocker; BUN, blood urea nitrogen; CI, confidence interval; CKD, chronic kidney disease; DKD, diabetic kidney disease; DPP, dipeptidyl peptidase; ESRD, end-stage renal disease; FBG, fasting blood glucose; GLP-1, glucagon-like peptide-1; HbA1c, glycosylated hemoglobin; KDOQI, National kidney foundation disease outcomes quality initiative; PRISMA, preferred reporting items for systematic reviews and meta-analyses; RAAS, renin angiotensin aldosterone system; RCTs, randomised controlled trials; Scr, serum creatinine; SGLT2is, glucose cotransporter 2 inhibitors; SMD, standardized mean difference; STZ, streptozotocin; TC, total cholesterol; TCM, traditional Chinese medicine; TG, triglyceride; 24-h UPRO, 24-h urinary protein.
