# Supplementary material for: Traditional Chinese medicine for the treatment of diabetic kidney disease: A study-level pooled analysis of 44 randomized controlled trials
Source: Front Pharmacol. 2022 Oct 13;13:1009571. doi: 10.3389/fphar.2022.1009571 (PMC9606328; doi:10.3389/fphar.2022.1009571)
Supplement: Supplementary file 1 [file DataSheet3.docx]

**Supplementary Tables.** Subgroup analyses

**Supplementary Table 1.** Subgroup analyses for the effects of FBG.

| **Variables** | **No. Studies** | **Sample Size** | **SMD** | **95%CI** | ***I*^2^ (%)** | ***P* for Interaction** |
| --- | --- | --- | --- | --- | --- | --- |
| Western medicine treatment |  |  |  |  |  | 0.39 |
| ACEI | 10 | 999 | -0.37 | -0.63 to -0.12 | 74 |  |
| ARB | 7 | 454 | -0.22 | -0.46 to -0.03 | 41 |  |
| Sample size |  |  |  |  |  | 0.87 |
| ≤70 | 9 | 486 | -0.29 | -0.51 to -0.07 | 31 |  |
| ＞70 | 11 | 1187 | -0.32 | -0.54 to -0.09 | 72 |  |
| Year of publication |  |  |  |  |  | 0.04 |
| Before 2015 | 12 | 913 | -0.24 | -0.43 to -0.05 | 49 |  |
| Year 2015 and after | 8 | 760 | -0.40 | -0.66 to -0.13 | 69 |  |
| Duration of treatment |  |  |  |  |  | 0.07 |
| ≤8 weeks | 5 | 430 | -0.14 | -0.38 to -0.11 | 35 |  |
| ＞8 weeks | 10 | 783 | -0.45 | -0.68 to -0.22 | 59 |  |
| Duration of DKD |  |  |  |  |  | 0.43 |
| ＜10 years | 7 | 498 | -0.30 | -0.48 to -0.12 | 0 |  |
| ≥10 years | 6 | 508 | -0.18 | -0.42 to 0.05 | 44 |  |
| Average age |  |  |  |  |  | 0.01 |
| ＜60 years | 14 | 1267 | -0.29 | -0.50 to -0.08 | 71 |  |
| ≥60 years | 6 | 406 | -0.34 | -0.54 to -0.14 | 0 |  |

Abbreviations: ACEI, angiotensin-converting enzyme inhibitor; ARB, angiotensin receptor blocker; CI, confidence interval; DKD, diabetes kidney disease;FBG, fasting blood glucose; SMD, standard mean difference.

**Supplementary Table 2..** Subgroup analyses for the effects of HbA1C.

| **Variables** | **No. Studies** | **Sample Size** | **SMD** | **95%CI** | ***I*^2^ (%)** | ***P* for Interaction** |
| --- | --- | --- | --- | --- | --- | --- |
| Western medicine treatment |  |  |  |  |  | 0.62 |
| ACEI | 10 | 999 | -0.68 | -1.01 to -0.27 | 90 |  |
| ARB | 7 | 454 | -0.53 | -0.98 to -0.08 | 82 |  |
| Sample size |  |  |  |  |  | 0.28 |
| ≤70 | 9 | 486 | -0.49 | -0.69 to -0.29 | 17 |  |
| ＞70 | 11 | 1187 | -0.74 | -1.15 to -0.33 | 91 |  |
| Year of publication |  |  |  |  |  | 0.91 |
| Before 2015 | 12 | 913 | -0.62 | -1.00 to -0.23 | 87 |  |
| Year 2015 and after | 8 | 760 | -0.65 | -1.00 to -0.29 | 82 |  |
| Duration of treatment |  |  |  |  |  | 0.53 |
| ≤8 weeks | 5 | 430 | -1.07 | -2.14 to -0.00 | 96 |  |
| ＞8 weeks | 10 | 783 | -0.55 | -0.73 to -0.36 | 36 |  |
| Duration of DKD |  |  |  |  |  | 0.16 |
| ＜10 years | 7 | 498 | -1.01 | -1.69 to -0.34 | 91 |  |
| ≥10 years | 6 | 508 | -0.45 | -0.84 to -0.06 | 79 |  |
| Average age |  |  |  |  |  | 0.43 |
| ＜60 years | 14 | 1267 | -0.54 | -0.08 to -0.28 | 80 |  |
| ≥60 years | 6 | 406 | -0.86 | -1.60 to -0.12 | 92 |  |

Abbreviations: ACEI, angiotensin-converting enzyme inhibitor; ARB, angiotensin receptor blocker; CI, confidence interval; DKD, diabetes kidney disease; HbA1C, glycosylated hemoglobin; SMD, standard mean difference.

**Supplementary Table 3.** Subgroup analyses for the effects of Scr.

| **Variables** | **No. Studies** | **Sample Size** | **SMD** | **95%CI** | ***I*^2^ (%)** | ***P* for Interaction** |
| --- | --- | --- | --- | --- | --- | --- |
| Western medicine treatment |  |  |  |  |  | ＜0.01 |
| ACEI | 9 | 842 | -1.96 | -2.75 to -1.61 | 96 |  |
| ARB | 6 | 409 | -0.23 | -0.47 to 0.00 | 30 |  |
| Sample size |  |  |  |  |  | 0.11 |
| ≤70 | 9 | 705 | -1.16 | -1.87 to -0.45 | 93 |  |
| ＞70 | 10 | 665 | -1.34 | -1.92 to -0.76 | 94 |  |
| Year of publication |  |  |  |  |  | 0.86 |
| Before 2010 | 9 | 785 | -1.77 | -2.60 to -0.94 | 96 |  |
| Year 2010 and after | 10 | 783 | -0.87 | -1.32 to -0.41 | 89 |  |
| Duration of treatment |  |  |  |  |  | 0.05 |
| ≤8 weeks | 6 | 468 | -0.80 | -1.24 to -0.36 | 81 |  |
| ＞8 weeks | 10 | 855 | -1.76 | -2.60 to -0.92 | 96 |  |
| Duration of DKD |  |  |  |  |  | 0.007 |
| ≤10 years | 6 | 577 | -2.81 | -4.08 to -1.54 | 97 |  |
| ＞10 years | 4 | 336 | -0.81 | -1.54 to -0.09 | 90 |  |
| Average age |  |  |  |  |  | 0.11 |
| ＜60 years | 12 | 854 | -1.28 | -1.95 to -0.61 | 69.9 |  |
| ≥60 years | 5 | 516 | -1.44 | -2.06 to -0.81 | 30.1 |  |

Abbreviations: ACEI, angiotensin-converting enzyme inhibitor; ARB, angiotensin receptor blocker; CI, confidence interval; DKD, diabetes kidney disease; Scr, serum creatinine; SMD, standard mean difference.

**Supplementary Table 4.** Subgroup analyses for the effects of BUN.

| **Variables** | **No. studies** | **Sample Size** | **SMD** | **95%CI** | ***I*^2^ (%)** | ***P* for Interaction** |
| --- | --- | --- | --- | --- | --- | --- |
| Western medicine treatment |  |  |  |  |  | 0.98 |
| ACEI | 9 | 842 | -0.53 | -0.97 to -0.10 | 89 |  |
| ARB | 6 | 409 | -0.54 | -1.12 to 0.03 | 87 |  |
| Sample size |  |  |  |  |  | ＜0.01 |
| ≤70 | 9 | 534 | -0.92 | -1.61 to -0.23 | 93 |  |
| ＞70 | 10 | 1034 | -0.63 | -1.02 to -0.24 | 89 |  |
| Year of publication |  |  |  |  |  | 0.97 |
| Before 2010 | 9 | 785 | -0.75 | -1.31 to -0.19 | 93 |  |
| Year 2010 and after | 10 | 783 | -0.77 | -1.21 to -0.32 | 89 |  |
| Duration of treatment |  |  |  |  |  | 0.27 |
| ≤8 weeks | 6 | 468 | -1.07 | -1.92 to -0.21 | 94 |  |
| ＞8 weeks | 10 | 855 | -0.54 | -0.94 to -0.14 | 87 |  |
| Duration of DKD |  |  |  |  |  | 0.64 |
| ＜10 years | 6 | 577 | -1.30 | -2.15 to -0.46 | 95 |  |
| ≥10 years | 4 | 336 | -1.01 | -1.93 to -0.09 | 93 |  |
| Average age |  |  |  |  |  | 0.13 |
| ＜60 years | 12 | 854 | -0.59 | -0.98 to -0.21 | 87 |  |
| ≥60 years | 5 | 516 | -1.44 | -2.47 to -0.41 | 96 |  |

Abbreviations: ACEI, angiotensin-converting enzyme inhibitor; ARB, angiotensin receptor blocker; BUN, blood urea nitrogen; CI, confidence interval; DKD, diabetes kidney disease; SMD, standard mean difference.

**Supplementary Table 5.** Subgroup analyses for the effects of TC.

| **Variables** | **No. Studies** | **Sample Size** | **SMD** | **95%CI** | ***I*^2^ (%)** | ***P* for Interaction** |
| --- | --- | --- | --- | --- | --- | --- |
| Western medicine treatment |  |  |  |  |  | 0.48 |
| ACEI | 11 | 1063 | -1.14 | -1.80 to -0.47 | 96 |  |
| ARB | 5 | 356 | -0.68 | -1.76 to -0.40 | 95 |  |
| Sample size |  |  |  |  |  | 0.12 |
| ≤70 | 7 | 369 | -0.40 | -1.39 to -0.60 | 95 |  |
| ＞70 | 11 | 1225 | -1.27 | -1.77 to -0.77 | 84 |  |
| Year of publication |  |  |  |  |  | 0.31 |
| Before 2010 | 5 | 547 | -1.44 | -2.63 to -0.25 | 97 |  |
| Year 2010 and after | 13 | 1047 | -0.77 | -1.28 to -0.26 | 93 |  |
| Duration of treatment |  |  |  |  |  | 0.10 |
| ≤8 weeks | 6 | 472 | -1.73 | -2.76 to -0.70 | 95 |  |
| ＞8 weeks | 8 | 724 | -0.85 | -1.45 to 0.24 | 96 |  |
| Duration of DKD |  |  |  |  |  | 0.06 |
| ≤5 years | 4 | 384 | -0.3 | -1.64 to 1.04 | 97 |  |
| ＞5 years | 7 | 609 | -1.79 | -2.57 to -1.02 | 94 |  |
| Average age |  |  |  |  |  | 0.0001 |
| ＜60 years | 14 | 1210 | -0.84 | -1.26 to -0.42 | 91 |  |
| ≥60 years | 4 | 384 | -1.36 | -3.37 to 0.64 | 98 |  |

Abbreviations: ACEI, angiotensin-converting enzyme inhibitor; ARB, angiotensin receptor blocker; CI, confidence interval; DKD, diabetes kidney disease; SMD, standard mean difference; TC, total cholesterol.

**Supplementary Table 6.** Subgroup analyses for the effects of TG.

| **Variables** | **No. Studies** |  | **Sample Size** | **SMD** | **95%CI** | ***I*^2^ (%)** | ***P* for Interaction** |
| --- | --- | --- | --- | --- | --- | --- | --- |
| Western medicine treatment |  |  |  |  |  |  | 0.31 |
| ACEI | 11 |  | 1063 | -1.50 | -2.35 to -0.65 | 97 |  |
| ARB | 5 |  | 356 | -0.80 | -1.87 to 0.28 | 95 |  |
| Sample size |  |  |  |  |  |  | ＜0.001 |
| ≤70 | 7 |  | 369 | -0.80 | -1.71 to 0.12 | 94 |  |
| ＞70 | 11 |  | 1225 | -1.41 | -2.17 to -0.64 | 97 |  |
| Year of publication |  |  |  |  |  |  | 0.39 |
| Before 2010 | 5 |  | 547 | -1.70 | -3.30 to -0.09 | 98 |  |
| Year 2010 and after | 13 |  | 1047 | -0.96 | -1.51 to -0.41 | 94 |  |
| Duration of treatment |  |  |  |  |  |  | 0.46 |
| ≤8 weeks | 6 |  | 472 | -1.73 | -2.86 to -0.60 | 96 |  |
| ＞8 weeks | 8 |  | 724 | -1.39 | -2.26 to 0.01 | 97 |  |
| Duration of DKD |  |  |  |  |  |  | 0.54 |
| ≤5 years | 4 |  | 384 | -0.93 | -3.21 to 1.34 | 99 |  |
| ＞5 years | 7 |  | 609 | -1.68 | -2.52 to -0.85 | 95 |  |
| Average age |  |  |  |  |  |  | 0.67 |
| ＜60 years | 14 |  | 1210 | -1.02 | -1.49 to -0.56 | 93 |  |
| ≥60 years | 4 |  | 384 | -1.60 | -4.16 to 0.97 | 96 |  |

Abbreviations: ACEI, angiotensin-converting enzyme inhibitor; ARB, angiotensin receptor blocker; CI, confidence interval; DKD, diabetes kidney disease; SMD, standard mean difference; TG, triglyceride.
