# Supplementary material for: Traditional Chinese medicine for the treatment of diabetic kidney disease: A study-level pooled analysis of 44 randomized controlled trials
Source: Front Pharmacol. 2022 Oct 13;13:1009571. doi: 10.3389/fphar.2022.1009571 (PMC9606328; doi:10.3389/fphar.2022.1009571)
Supplement: Supplementary file 2 [file DataSheet2.docx]

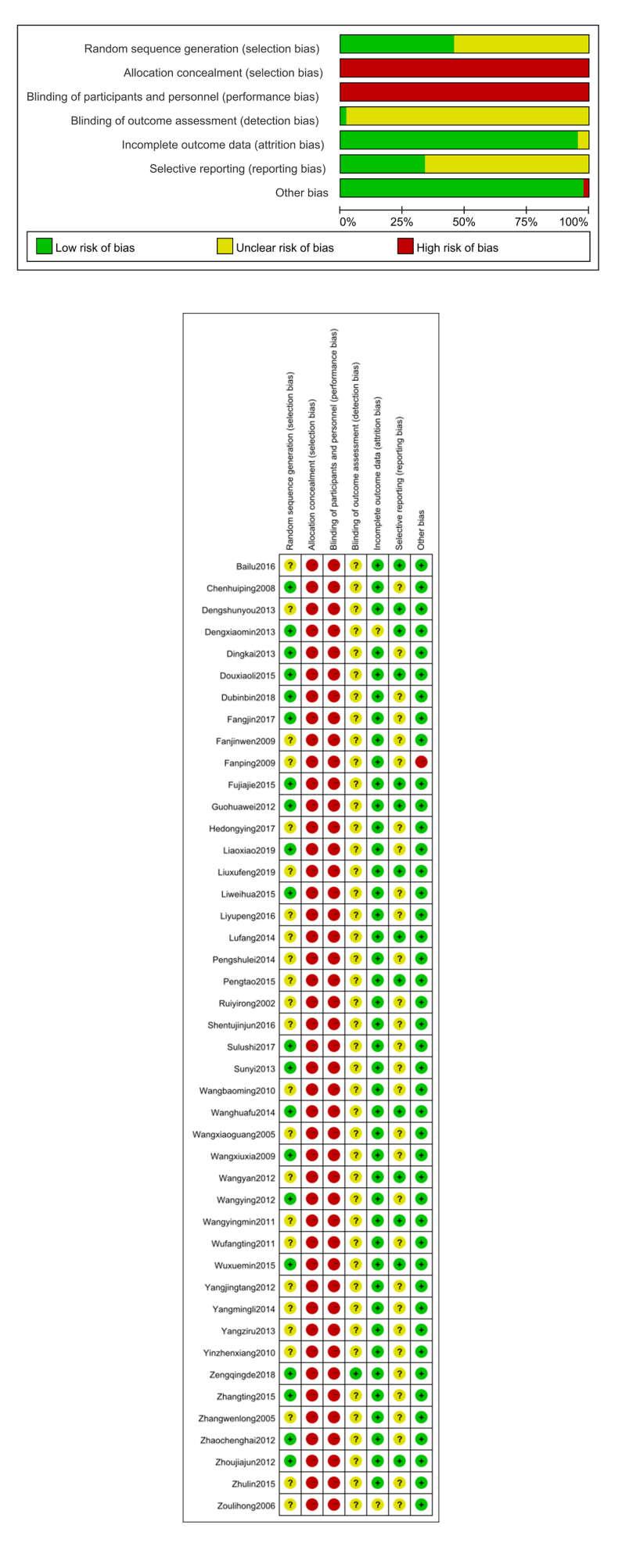


**Supplementary Figure 1.** Risk of bias for the included trials.

**
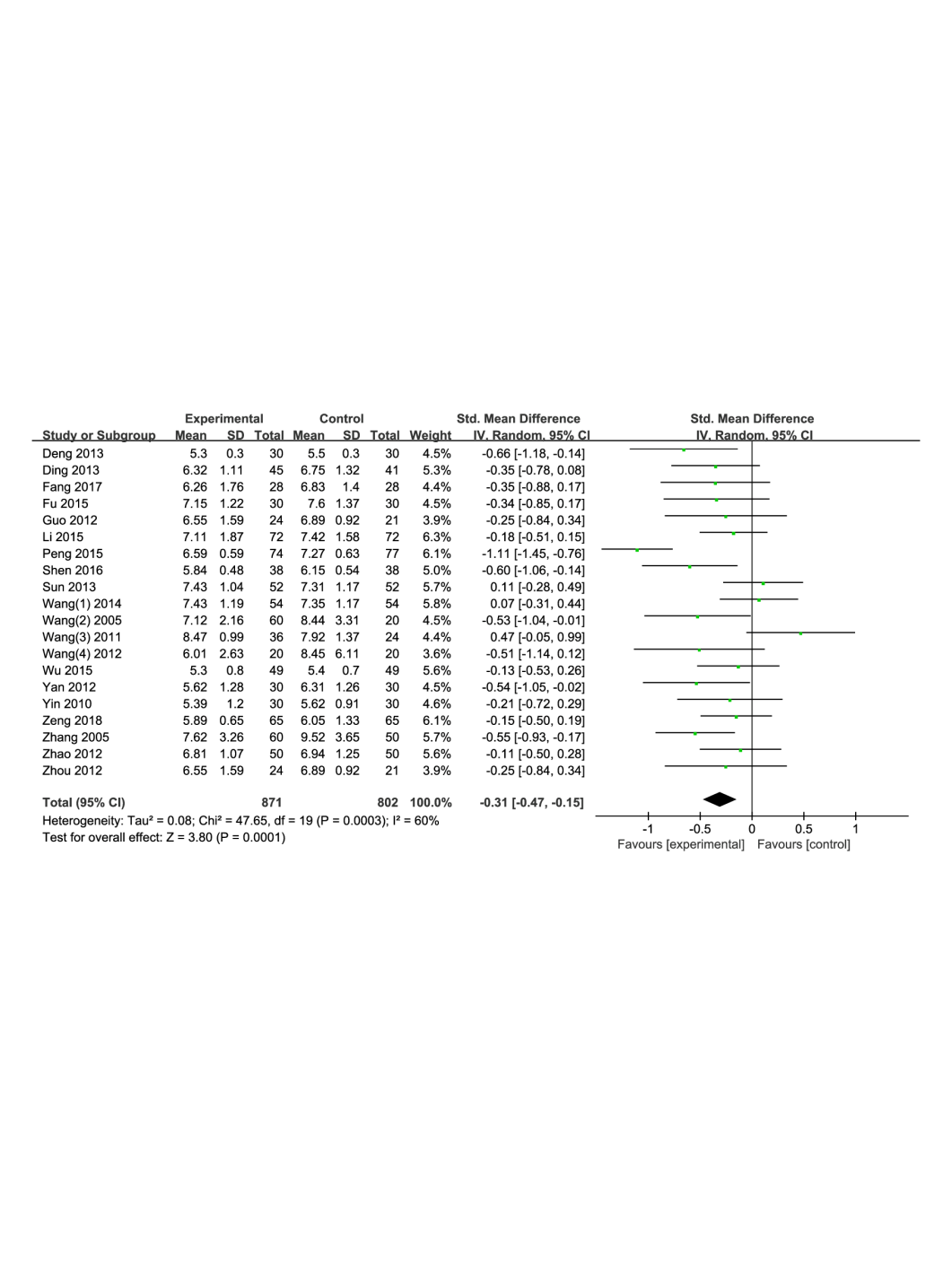
**

**Supplementary Figure 2.** Forest plot for meta-analysis of the effect of TCM combined with western medicine on FBG.


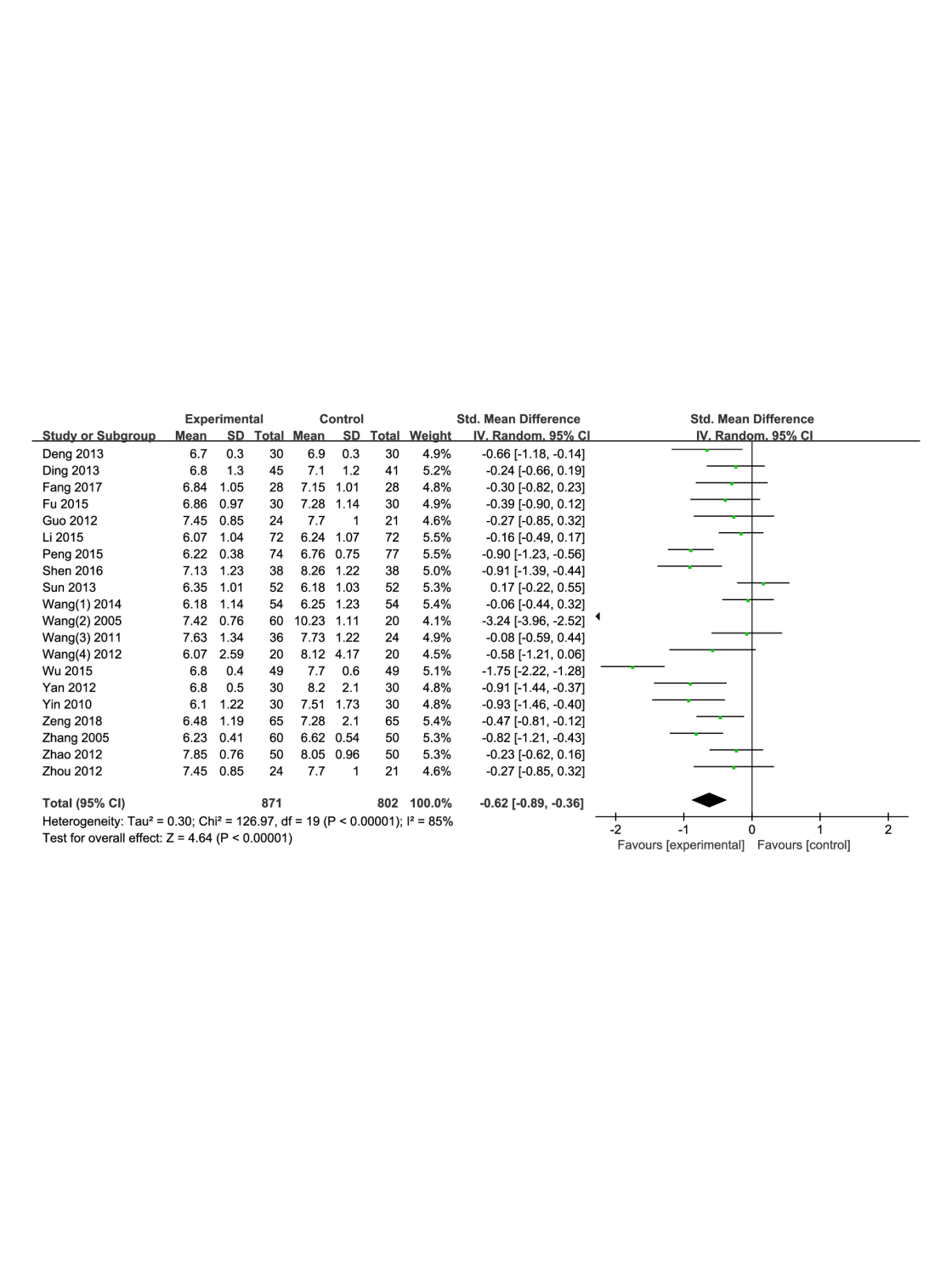


**Supplementary Figure 3.** Forest plot for meta-analysis of the effect of TCM combined with western medicine on HbA1c.


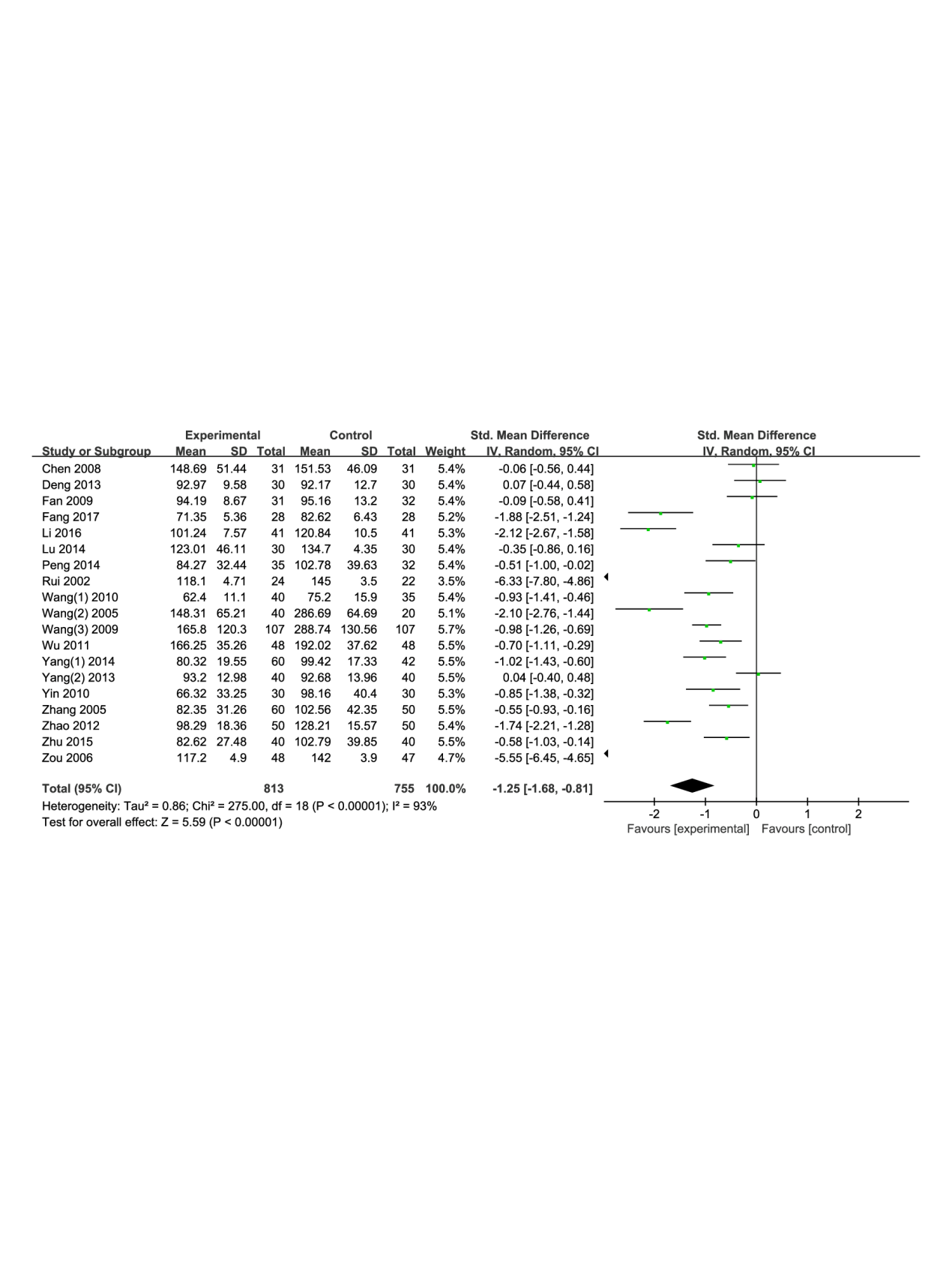


**Supplementary Figure 4.** Forest plot for meta-analysis of the effect of TCM combined with western medicine on Scr.


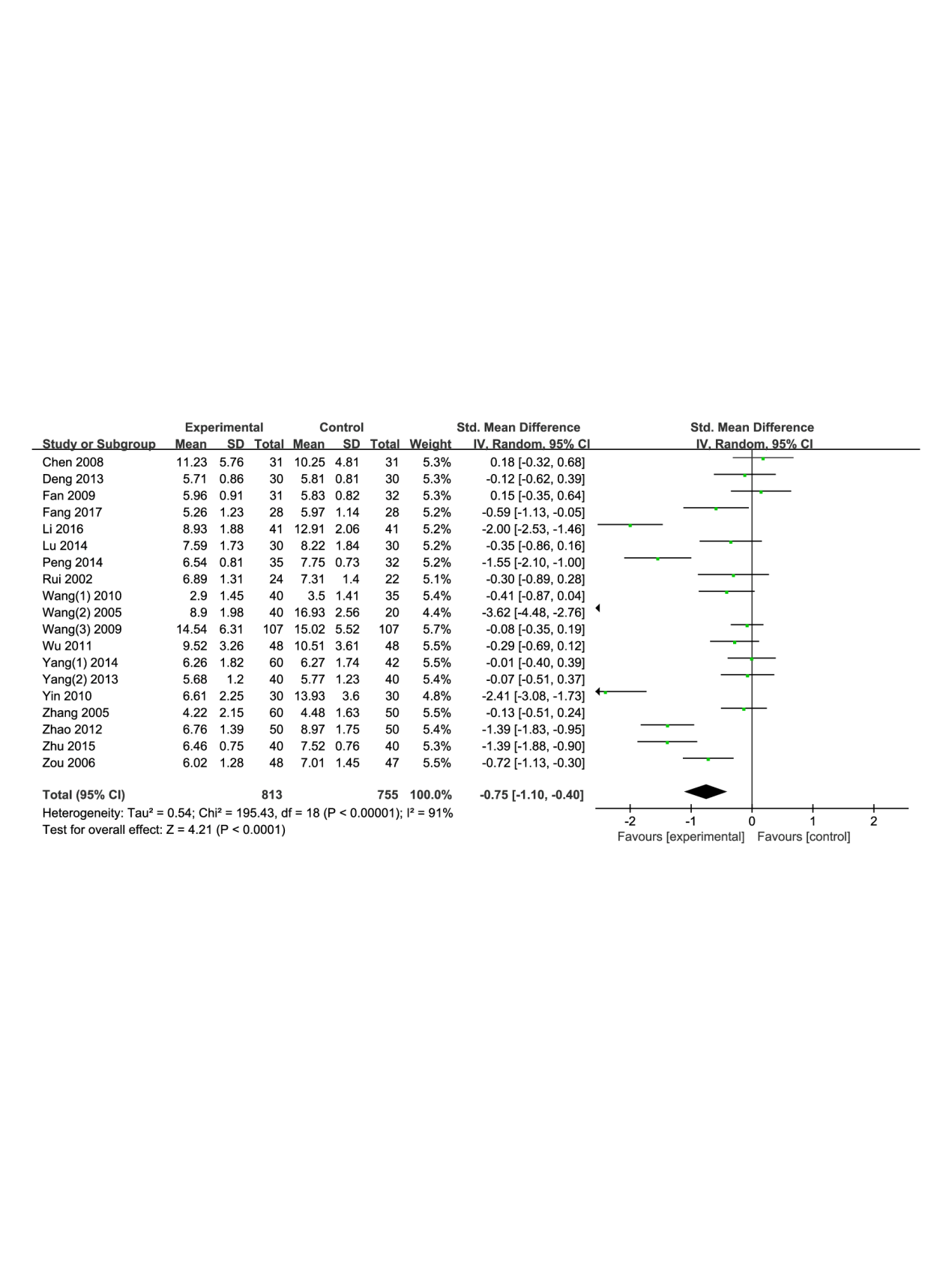


**Supplementary Figure 5.** Forest plot for meta-analysis of the effect of TCM combined with western medicine on BUN.


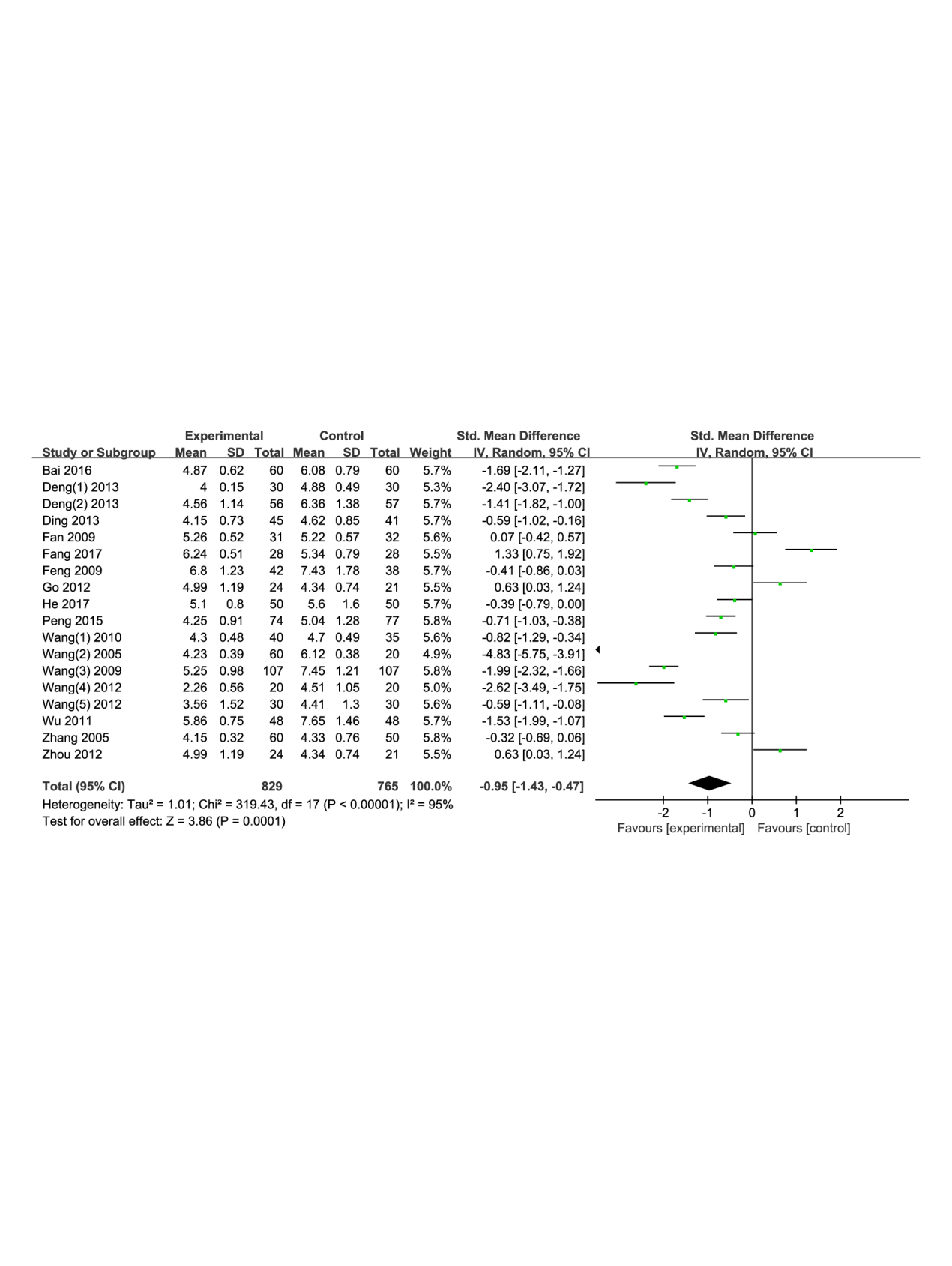


**Supplementary Figure 6.** Forest plot for meta-analysis of the effect of TCM combined with western medicine on TC.


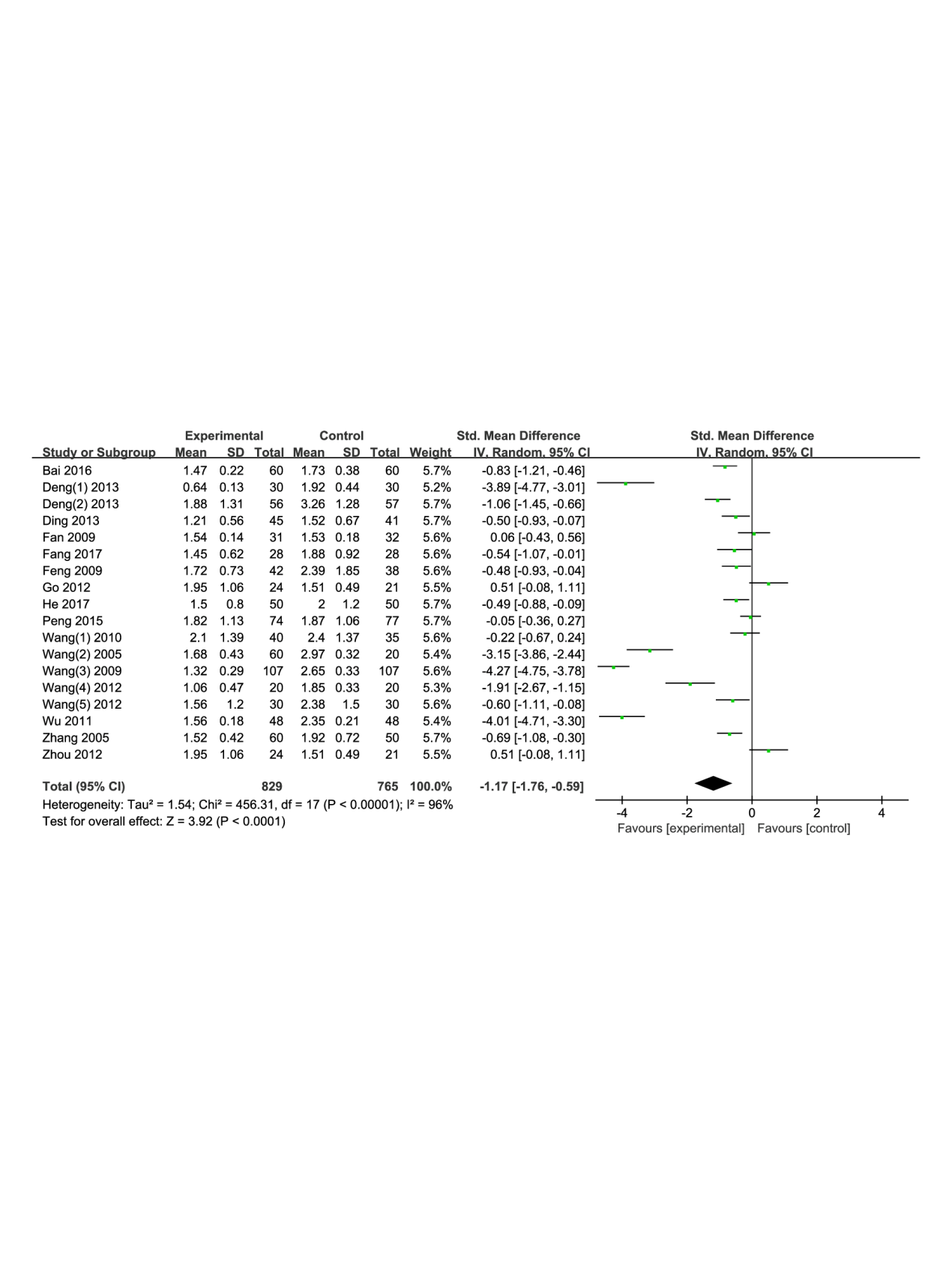


**Supplementary Figure 7.** Forest plot for meta-analysis of the effect of TCM combined with western medicine on TG.

.
