# Supplementary material for: Traditional Chinese medicine for the treatment of diabetic kidney disease: A study-level pooled analysis of 44 randomized controlled trials
Source: Front Pharmacol. 2022 Oct 13;13:1009571. doi: 10.3389/fphar.2022.1009571 (PMC9606328; doi:10.3389/fphar.2022.1009571)
Supplement: Supplementary file 3 [file DataSheet1.docx]

**Supplementary Methods**

Text S1 Search strategy

**Database: Pubmed**

**Search Strategy:**

--------------------------------------------------------------------------------

***Traditional Chinese medicine terms:***

1 "Medicine, Chinese Traditional"[Mesh]

2 (traditional chinese medicine* or chinese traditional medicine* or chinese herbal drugs* or medicine,

traditional* or Medicine, Ayurvedic* or ethnomedicine* or ethnobotany* or medicine, kampo* or

kanpo* or TCM* or phytotherapy* or herbology* or plant preparation* or plant extract* or plants,

medicine* or materia medica* or single prescription* or chinese medicine herb* or herbal medicine*

or herbs*) [Title/Abstract]

3 or/1-2

***Diabetic kidney disease terms:***

4 "Diabetic Nephropathies"[Mesh]

5 (diabetic kidney disease* or diabetic kidney diseases* or diabetic nephropathy* or kimmelstiel

wilson syndrome* or kimmelstiel wilson disease* or diabetic glomerulosclerosis* or nodular

glomerulosclerosis* or intracapillary glomerulosclerosis* or albuminuria* or microalbuminuria*

or proteinuria* or glomerulosclerosis* or Glomerulonephritis* or Kimmelstiel wilson nephropathy*

or diabetic nephrosclerosis*) [Title/Abstract]

6 or/4-5

***Randomized controlled trial terms:***

7 "Randomized controlled trial"[Publication Type] or "controlled clinical trial"[Publication Type]

8 (randomized controlled trial* or controlled clinical trial * or randomized* or placebo * or drug therapy * or randomly * or trial* or groups*)[Title/Abstract]

9 or/7-8

***Final search results: Traditional Chinese Medicine terms and Diabetic Kidney Disease terms and Randomized controlled trial terms:***

10 3 and 6 and 9 (46)

Text S2 Search strategy

**Database: EMBASE**

**Search Strategy:**

--------------------------------------------------------------------------------

***Traditional Chinese medicine terms:***

1 'chinese medicine'/exp

2 'herbal medicine'/exp

3 'herbs'/exp

4 (traditional chinese medicine* or chinese traditional medicine* or chinese herbal drugs* or chinese

drugs, plant* or medicine, traditional* or ethnopharmacology* or ethnomedicine* or ethnobotany* or

medicine, kampo* or kampo medicine* or tcm* or medicine, ayurvedic* or phytotherapy* or

herbology* or medicinal plant* or plant extract* or plants, medicine* plant preparation* or materia

medica* or single prescription* or chinese medicine herb*):ab,ti

5 or/1-4

***Diabetic kidney disease terms:***

4 'diabetic nephropathy'/exp

5 (‘diabetic nephropathies*’ or ‘diabetic nephropathy*’ or ‘diabetic kidney disease*’ or ‘diabetic kidney diseases*’ or ‘kimmelstiel wilson syndrome*’ or ‘kimmelstiel wilson disease*’ or ‘diabetic glomerulosclerosis*’ or ‘nodular glomerulosclerosis*’ or ‘intracapillary glomerulosclerosis*’ or albuminuria* or microalbuminuria* or proteinuria* or glomerulonephritis* or glomerulosclerosis* or ‘diabetic nephrosclerosis*’):ab,ti

6 or/4-5

***Randomized controlled trial terms:***

7 'randomized controlled trial'/exp

8 (‘randomized controlled trial*’ or ‘controlled clinical trial *’ or randomized* or placebo * or ‘drug therapy *’ or randomly * or trial* or groups*):ab,ti

9 or/7-8

***Final search results: Traditional Chinese Medicine terms and Diabetic Kidney Disease terms and Randomized controlled trial terms:***

10 5 and 6 and 9 (401)

Text S3 Search strategy

**Database: Cochrane Library**

**Search Strategy:**

--------------------------------------------------------------------------------

***Traditional Chinese medicine terms:***

1 MeSH descriptor: [Medicine, Traditional] explode all trees

2 (chinese drugs, plant* or medicine, traditional* or ethnopharmacology* or ethnomedicine* or

ethnobotany* or medicine, kampo* or kanpo* or tcm* or medicine, ayurvedic* or phytotherapy* or

herbology* or plants, medicinal* or plant preparation* or plant extract* or plants, medicine* or materia

medica* or single prescription* or word variations have been searched* or chinese medicine herb* or

herbal medicine* or herbs*):ti,ab,kw (word variations have been searched)

3 or/1-2

***Diabetic kidney disease terms:***

4 MeSH descriptor: [Diabetic Nephropathies] explode all trees

5 (diabetic kidney disease* or diabetic kidney diseases* or diabetic nephropathy* or kimmelstiel

wilson syndrome* or kimmelstiel wilson disease* or diabetic glomerulosclerosis* or nodular

glomerulosclerosis* or intracapillary glomerulosclerosis* or albuminuria* or microalbuminuria*

or proteinuria* or glomerulosclerosis* or Glomerulonephritis* or Kimmelstiel wilson nephropathy*

or diabetic nephrosclerosis*):ti,ab,kw (word variations have been searched)

6 or/4-5

***Randomized controlled trial terms:***

10 "Randomized controlled trial"[Publication Type] or "controlled clinical trial"[Publication Type]

11 (randomized controlled trial* or controlled clinical trial * or randomized* or placebo * or drug therapy * or randomly * or trial* or groups*):ti,ab,kw (Word variations have been searched)

12 or/10-17

***Final search results: Combining Cancer and Stroke and Study design:***

13 3 and 6 and 12 (232)

Text S4 Search strategy

**Database: Chinese BioMedical Literature Database**

**Search Strategy**

--------------------------------------------------------------------------------

***中医药 terms:***

1 "中草药"[不加权:扩展]

2 （药用植物* or植物疗法* or中草药* or中药* or中国草药* or中国传统草药* or植物药* or中医内治法* or草药* or草本药物* or中医药)[常用字段:智能]

3 or/1-2

***糖尿病肾病 terms:***

4 "糖尿病肾病"[不加权:扩展]

5 （糖尿病肾病* or K-W结节* or结节性肾小球硬化症* or糖尿病肾小球硬化症* or糖尿病性肾小球硬化症* or糖尿病性弥漫性肾小球硬化症* or糖尿病性肾病)[常用字段:智能]

6 or/4-5

***随机对照试验 terms:***

***7*** 随机 ?

8 "随机对照试验"[不加权:扩展]

9 "随机对照试验(主题)"[不加权:扩展]

10 or/7-9

***Final search results: Combining 中医药 and 糖尿病肾病 and 随机对照试验:***

13 3 and 6 and 12 (2102)
